# IL-6 contributes to metastatic switch via the differentiation of monocytic-dendritic progenitors into prometastatic immune cells

**DOI:** 10.1136/jitc-2021-002856

**Published:** 2021-06-17

**Authors:** Ksenia Magidey-Klein, Tim J Cooper, Ksenya Kveler, Rachelly Normand, Tongwu Zhang, Michael Timaner, Ziv Raviv, Brian P. James, Roi Gazit, Ze'ev A. Ronai, Shai Shen-Orr, Yuval Shaked

**Affiliations:** 1 Faculty of Medicine, Technion Israel Institute of Technology, Haifa, Israel; 2 Division of Cancer Epidemiology & Genetics, National Cancer Institute, National Institute of Health, Bethesda, Maryland, USA; 3 Cancer Center, Sanford Burnham Prebys Medical Discovery Institute, La Jolla, California, USA; 4 Department for Microbiology, Immunology and Genetics, Ben-Gurion University of the Negev, Beer-Sheva, Southern, Israel

**Keywords:** immunity, cellular, immunomodulation, macrophages, melanoma

## Abstract

**Background:**

Metastasis is the major cause of death in patients with cancer. Myeloid skewing of hematopoietic cells is a prominent promoter of metastasis. However, the reservoir of these cells in the bone marrow (BM) compartment and their differentiation pattern from hematopoietic stem and progenitor cells (HSPCs) have not been explored.

**Methods:**

We used a unique model system consisting of tumor cell clones with low metastatic potential or high metastatic potential (met-low and met-high, respectively) to investigate the fate of HSPC differentiation using murine melanoma and breast carcinoma. Single-cell RNA sequencing (scRNA-seq) analysis was performed on HSPC obtained from the BM of met-low and met-high tumors. A proteomic screen of tumor-conditioned medium integrated with the scRNA-seq data analysis was performed to analyze the potential cross talk between cancer cells and HSPCs. Adoptive transfer of tumor-educated HSPC subsets obtained from green fluorescent protein (GFP)+ tagged mice was then carried out to identify the contribution of committed HSPCs to tumor spread. Peripheral mononuclear cells obtained from patients with breast and lung cancer were analyzed for HSPC subsets.

**Results:**

Mice bearing met-high tumors exhibited a significant increase in the percentage of HSPCs in the BM in comparison with tumor-free mice or mice bearing met-low tumors. ScRNA-seq analysis of these HSPCs revealed that met-high tumors enriched the monocyte-dendritic progenitors (MDPs) but not granulocyte-monocyte progenitors (GMPs). A proteomic screen of tumor- conditioned medium integrated with the scRNA-seq data analysis revealed that the interleukin 6 (IL-6)–IL-6 receptor axis is highly active in HSPC-derived MDP cells. Consequently, loss of function and gain of function of IL-6 in tumor cells resulted in decreased and increased metastasis and corresponding MDP levels, respectively. Importantly, IL-6-educated MDPs induce metastasis within mice bearing met-low tumors—through further differentiation into immunosuppressive macrophages and not dendritic cells. Consistently, MDP but not GMP levels in peripheral blood of breast and lung cancer patients are correlated with tumor aggressiveness.

**Conclusions:**

Our study reveals a new role for tumor-derived IL-6 in hijacking the HSPC differentiation program toward prometastatic MDPs that functionally differentiate into immunosuppressive monocytes to support the metastatic switch.

## Background

Despite recent improvements in various cancer therapies, overt systemic metastatic disease remains mostly incurable, accounting for the majority of cancer-related mortalities. Metastasis is a multistep process that involves dissemination of cancer cells from the primary tumor mass, intravasation into blood circulation, seeding, and subsequent growth at distant sites.^1^ Successful colonization of tumor cells at distant sites relies, in part, on bone marrow-derived cells (BMDCs) that are recruited earlier than tumor cells to distant organ sites where they contribute to the formation of a premetastatic niche.^2 3^ Among these BMDCs are immunosuppressive myeloid cells such as M2 macrophages and myeloid-derived suppressor cells (MDSCs).^4 5^

All immune cells within tumors originate from hematopoietic stem and progenitor cells (HSPCs). HSPCs are sensitive to external stimuli, such as infection or injury. In response, they enter an extensive proliferative phase to increase the reservoir of required immune cells, shifting the balance between various populations of leucocytes. Once the stimulus ceases, HSPCs return to a quiescent stage, maintaining a normal homeostasis.^6^ Tumors also secrete external stimuli, which contribute to HSPC differentiation. It is well established that tumors induce myelopoiesis as a means of supplying immature progenitors that act as immunosuppressive cells. For example, it has been demonstrated that the composition of circulating HSPCs is significantly altered in patients with solid tumors, with increased levels of granulocyte-monocyte progenitors (GMPs) and a general bias toward granulocyte formation, compared with healthy patients.^7^ In addition, elevated numbers of circulating HSPCs correlate with tumor aggressiveness and decreased overall survival.^7 8^ In the context of HPSC differentiation, it has been previously thought that GMPs are differentiated into neutrophils or monocyte-dendritic progenitors (MDPs), which further differentiate into monocytes and dendritic cells (DCs).^9^ However, a recent study demonstrated that GMPs and MDPs produce functionally distinct monocytes. While GMPs produce neutrophils and monocytes, MDPs are differentiated into monocytes and DCs but not neutrophils.^10^ The contribution of these progenitors to external stimuli has been studied in the context of microbial stimuli; however, their differentiation pattern and contribution to tumor aggressiveness and metastasis are not known.

While the majority of studies focus on tumor education of the bone marrow (BM) niche and tumor-induced alterations of the HSPC differentiation program relative to non-tumor-bearing subjects, an equivalent comparison between high metastatic and low metastatic tumors has yet to be made. Here we show, for the first time, that high metastatic tumors regulate HSPC fate, shifting their differentiation pattern toward tumor and metastasis-supporting cell by promoting the MDP-derived lineage over that of the GMP-derived lineage. Specifically, we demonstrate that tumor-derived interleukin 6 (IL-6) expressed by high metastatic cells mediates a tumor–BM cross talk, which in turn leads to increased levels of MDPs. Importantly, these tumor-educated MDPs further give rise to an immunosuppressive macrophage phenotype rather than DCs, which ultimately promotes metastasis.

## Methods

### Tumor cell cultures

The 4T1 murine breast cancer cell line and B16-F1 and B16-F10 murine melanoma cell lines were purchased from the American Type Culture Collection (Manassas, VA, USA). Cells were used within 6 months of resuscitation. 67NR murine breast cancer cells were kindly provided by Prof. Sleeman (Menheim, Germany). 4T1 and 67NR cell lines have the same genetic background and represent cells with high metastatic potential (met-high) and low metastatic potential (met-low), respectively, as previously described.^11^ The cells were grown in Dulbecco’s modified Eagle’s medium (Sigma-Aldrich, Rehovot, Israel). B16-F10 and B16-F1 cells represent cells with met-high and met-high, respectively, as previously described.^12^ For overexpression of IL-6, B16-F1 cells were transfected with 1 µg DNA of pCMV3 vector encoding IL-6 or the empty control pCMV3 vector (EV) using PolyJet (SignaGen Laboratories, Frederick, MD, USA) following the manufacturer’s instructions. For generating stable clones, transfected cells were grown for 2 weeks with hygromycin (250 µg/mL). Conditioned medium obtained after 24 hours was analyzed for IL-6 levels using ELISA. Cell proliferation of IL-6-overexpressing cells was carried out by cell proliferation kit (XTT sodium slat), as previously described.^13^ Cells were grown in Roswell Park Memorial Institute medium (Sigma-Aldrich) and supplemented with 1.5% non-essential amino acids, 0.75% minimum essential medium (MEM vitamins, and 1.5% sodium bicarbonate. All cell media were supplemented with 5% fetal calf serum, 1% L-glutamine, 1% sodium pyruvate, and 1% Pen-Strep-Neomycin in solution (purchased from Biological Industries, Israel). The cells were cultured in a humidified chamber in 5% CO_2_ at 37°C. Cells were routinely tested for mycoplasma and found to be mycoplasma-free.

### Animals

Female BALB/c and C57BL/6 mice (8–10 weeks of age) were purchased from Envigo (Israel) or the Jackson Laboratory (Bar Harbor, ME, USA). For BM transplantation, co-inoculation, and adoptive transfer experiments, transgenic C57BL/6 mice expressing enhanced GFP under the regulation of the ubiquitin promoter were used (B6-EGFP; Jackson Laboratory). The Animal Care and Use Committees of the Technion (Haifa, Israel) and Sanford Burnham Prebys (San Diego, CA, USA) approved all animal studies and experimental protocols.

### Murine tumor models

4T1 and 67NR cells (5×10^5^ cells in 50 µL of serum-free medium) were orthotopically implanted in the mammary fat pad of BALB/c female mice. B16-F1 and B16-F10 cells (5×10^5^ cells in 200 µL of serum-free medium) were subdermally implanted into the flanks of C57BL/6 mice. Tumor volume was measured twice a week with Vernier calipers and calculated according to the formula width^2^×height×0.5. When the tumor size reached approximately 1500 mm^3^ (end point), mice were sacrificed and tumor, lungs, and femurs were removed. Blood was drawn by cardiac puncture prior to mice euthanasia.

In some experiments, mice were treated with anti-IL-6 antibodies (20 mg/kg, MP5-20F3 clone; BioXCell, Lebanon, NH, USA) or IgG control antibodies (20 mg/kg, MP5-20F3 clone), two times a week, as previously described.^12^ Twenty-four hours after the last injection, the mice were sacrificed for further pathological and cellular analysis.

In co-inoculation experiments, Lin-Sca1+Kit+ (LSK, considered as HSPCs) cells (25,000 cells/mouse) obtained from the BM of tumor-free GFP-expressing mice were mixed with B16-F1 or B16-F10 cells in Matrigel (BD Biosciences, San Jose, CA, USA) and subdermally implanted into the flanks of C57BL/6 mice. LSK cells mixed with Matrigel alone were used as control groups to evaluate spontaneous differentiation in Matrigel plugs. Lin− cells were purified using the MagCellect Mouse Hematopoietic Cell Lineage Depletion Kit (R&D Systems, Minneapolis, MN, USA) and immunostained for LSK markers. LSK cells were sorted using FACSAria IIIu (BD Biosciences). Mice were sacrificed 10 days after implantation, and tumors were removed.

### Human samples

Peripheral blood mononuclear cells (PBMCs) were obtained from patients with breast and lung cancer segregated into early and advanced tumor stages, before they undergo any therapy. The samples were obtained from the Rambam Healthcare Campus BioBank (Midgam, Haifa). Samples were collected under institutional ethics board (RMB-0631-17) after patients signed an informed consent. The clinical stages were classified based on tumor stage. In breast cancer (n=18 samples), stages T0–T1 were considered early stage (n=9) and stages T2–T4 were considered advanced stage (n=9). In lung cancer (n=17 samples), stages T0–T2 were considered early stage (n=8) and stages T3–T4 were considered advanced stage (n=9). Patient characteristics are indicated in online supplemental table S2.

10.1136/jitc-2021-002856.supp2 Supplementary data

### Flow cytometry acquisition and analysis

Lungs and tumors were prepared as single-cell suspensions as previously described.^13^ BM cells were flushed from the BM, and peripheral blood was collected either by retro-orbital bleed or by cardiac puncture. Cells were immunostained with specific antibodies against surface markers to define various cell types listed in online supplemental table S3. Conjugated monoclonal antibodies were purchased from BD Biosciences or BioLegend (Biolegend Way, San Diego, CA, USA). Sca1(D7)-PE/BV786, CD117(2B8)-APC, CD34(HM34)-PE, IL-7R(A7R34)-PE-Cy7, Flt3(A2F10)-PE-Cy5, F4/80(BM8)-PE, CD11b(M1/70)-PerCP, Gr-1(RB6 8C5)-BV510, FCγR(93)-BV510, CD115(AFS98)-PeCy7, CD206(C068C2)-BV421, CD11c(N418)-APC-Cy7, Ly6C(1A8)-BV605, Ly6G(HK1.4)-BV510, CD3ε(30-F11)-Alexa Fluor 700, B220(RA3-6B2)-BV605, and lineage cocktail (17A2/RB6-8C5/RA3-6B2/Ter 119/M1/70)-Alexa Fluor 700 or BV421. For the detection of human MDP or GMP in peripheral blood, peripheral blood mononuclear cells were conjugated with the antibodies (OKT3/M5E2/3G8/HIB19/2H7/HCD56)-BV510, CD38(HIT2)-PE, CD34(561)-BV421, CD123(6H6)-BV785, CD45RA(HI100)-APC, CD115(9-4D2-1E4)-Pe-Cy7 to detect MDPs and GMPs. For phospho-STAT3 (p-STAT3) analysis, naïve BM cells were stimulated first with escalating doses of IL-6 for 30 min at 37°C and subsequently were immunostained for MDPs. Right after, the cells were fixed in 1.6% paraformaldehyde and permeabilized in ice-cold 90% ethanol. Cells were immunostained with p-STAT3 (Tyr705)-AF488 at 4°C for 40 min. At least 500,000 events were acquired for each sample using BD LSRFortessa cytometer and analyzed with FlowJo V.10.2 software (Ashland, OR).

### BM transplantation

Donor GFP-expressing mice were inoculated with either B16-F1 or B16-F10 cancer cells. At end point, the mice were sacrificed and BM cells were obtained by flushing the femur and tibia with phosphate-buffered saline (PBS). Lineage depletion kit (R&D systems) was used to purify immature cells. Enriched cells were immunostained and sorted for LSK (5×10^3^ cells) and subsequently intravenously transplanted into recipient lethally irradiated mice (10 Gy in 8 min, single dose) together with supportive total BM cells (1×10^6^ cells/mouse), obtained from naïve (non-GFP) C57BL/6 mice. Recipient mice exhibiting less than 1% total chimerism were considered as failed transplantations and excluded from the analysis. Reconstitution recovery of GFP+ donor cells was tested after 16 weeks by collecting the blood from the retro-orbital venous plexus.

### MDP and GMP adoptive transfer

GFP-expressing mice were implanted with B16-F10 tumor cells and then treated with anti-IL-6 or IgG control antibodies, as described above. Lin− cells were purified from the BM using a lineage depletion kit (R&D systems) and immunostained for MDP or GMP sorting. MDPs (1×10^3^ cells) or GMPs (1×10^3^ cells) were intravenously injected to naïve C57BL/6 mice, which were subsequently implanted with B16-F1 tumor cells. When the tumors reached end point, the mice were sacrificed and MDP or GMP differentiation was evaluated by analyzing the GFP+ cells using flow cytometry. Lungs were harvested and subjected to histopathological staining for metastasis scoring.

### Mass cytometry acquisition and analysis

High-throughput mass cytometry (cytometry by time of flight (CyTOF)) analysis was performed as previously described.^14^ Briefly, tumors were prepared as single-cell suspensions. Cells were pooled (3×10^6^) and immunostained with a mixture of metal-tagged antibodies using the different surface markers as indicated in online supplemental table S4. All antibodies were conjugated using the MAXPAR reagent (Fluidigm, South San Francisco, CA, USA) and tittered prior to staining. Rhodium and iridium intercalators were used to identify live/dead cells. Cells were washed twice with PBS, ﬁxed in 1.6% formaldehyde (Sigma-Aldrich), washed again in ultrapure H_2_O, and acquired by CyTOF mass cytometry system (Fluidigm). The acquired data were uploaded to the Cytobank web server (Cytobank). CD11b+ myeloid live cells were used for the analysis, and the gated cells were segregated into subpopulation clusters by expression markers. Data analysis was performed by viSNE algorithm,^15^ via the Cytobank server. Changes in specific populations were validated by flow cytometry.

### Colony-forming assay

For mouse colony-forming units (CFUs), red blood cells were lysed from the peripheral blood of tumor-free or tumor-bearing mice. The cells were then seeded in triplicates at a concentration of 100,000 cells/well into six-well culture plates with M3434 methylcellulose (Methocult; Stem Cell Technologies, Vancouver, Canada). For assessing the differentiation pattern of naïve BM cells, MDPs, and GMPs in the presence of tumor-derived conditioned medium (TCM), incomplete methylcellulose medium M3431 (Stem Cell Technologies) was supplemented with 30% TCM obtained from 5×10^6^ total tumor cells after they were adjusted to culture for 24 hours in serum-free medium. BM cells (10,000 cells/well) and sorted MDP and GMP cells (50 cells/well) were plated for 12 days. Plates were imaged with a Zeiss microscope and colonies were scored.

### Single-cell RNA sequencing and data analysis

LSK cells were sorted from the BM of met-low and met-high melanoma tumor-bearing mice into two 384-well plates (Biorad Laboratories), one for each group and containing reverse transcriptase (RT) mix and barcoded 3′ RT primer, in nuclease-free water. The plates were kept at −80°C until analyzed. Single-cell RNA sequencing (scRNA-seq) was performed at the New York University Genome Center. On the day of library preparation, plates were removed from −80°C and placed in the thermal cycler for thaw, lyse, and annealing purposes (hold at 22°C, 22°C for 2 min, 72°C for 3 min, hold at 4°C). After the program ended, 2 µL of RT Mix 2 (each reaction consisting of 0.5 µL of 10 µM 5′ Custom TSO Primer (IDT Technologies), 0.925 µL of 5 M Betaine (ThermoFisher Scientific), 0.4 µL of 100 mM MgCl_2_ (Sigma-Aldrich), 0.125 µL of 20 U/µL Superase In RNase Inhibitor (Invitrogen), and 0.05 µL of 200 U/µL Maxima H Minus RT enzyme) was added to each well and samples were sealed and then mixed using the Eppendorf thermomixer at 2000 rpm for 30 s at room temperature. The plates were briefly centrifuged at 2000 rpm for 30 s at 4°C. Plates were then placed in the thermal cycler for the Maxima_RT program (hold at 50°C, 50°C for 94 min, 85°C for 5 min, hold at 4°C). After the RT program, each sample was treated with 7 µL of complementary DNA (cDNA) PCR Master mix (each reaction consisting of 0.25 µL of 10 µM IS-PCR Primer Mix (IDT Technologies), 0.5 µL of molecular grade water, and 6.25 µL of 2× KAPA Hifi ReadyMix (Roche)). Samples were mixed on the thermomixer at 2000 rpm for 30 s at room temperature and centrifuged briefly at 2000 rpm for 30 s at 4°C. Plates were placed in the thermal cycler for three-step cDNA amplification (hold at 98°C, 13 cycles of (98°C for 15 s; 75°C for 20 s; 72°C for 6 min), 5 cycles of (98°C for 15 s; 72°C for 20 s; 72°C for 6 min), 13 cycles of (98°C for 15 s; 67°C for 20 s; 72°C for 6 min), 72°C for 5 min, hold at 4°C). After cDNA amplification, samples were stored at 4°C until ready for cDNA pooling and cleanup. The pools were first cleaned with a 0.7× SPRISelect bead cleanup followed by a 0.6× SPRISelect bead cleanup and eluted in 25 µL of elution buffer. Pooled cDNA was quantified with Qubit HS DNA (Invitrogen) and Fragment Analyzer HS-NGS (Agilent). Pooled and purified cDNA (600 pg) was taken into Nextera XT (Illumina) library prep and tagmented according to the manufacturer’s instructions. The samples were given custom P7 and P5 adapters (10 µM, 1 µL for each adapter) and amplified using a modified NXT scRNA PCR program (hold at 95°C, 95°C for 30 s, 13 cycles of (95°C for 10 s, 55°C for 30 s, 72°C for 30 s), 72°C for 5 min, hold at 4°C). Each final library was quantified with Qubit HS DNA (Invitrogen) and Fragment Analyzer HS-NGS (Agilent), and then diluted to 2 nM. The libraries were loaded at a concentration of 8.5 pM on a HiSeq Rapid Run with run cycles of 26×9×9 ×76 and were compiled into a digital gene expression matrix used for downstream analyses.

For data filtering and clustering, Seurat R package V.3.00^16^ was used. Cells with less than 1000 non-zero genes were filtered out; genes expressed in less than three cells and non-coding genes were omitted. Outlier cells with more than 5% of expressed genes coming from mitochondria were removed from the analysis, as high mitochondrial expression likely indicates cells undergoing apoptosis. Altogether, the filtered data contained 758 cells and 13,997 genes. Following log-normalization, the top 2000 variable genes were identified and used to find Louvain clusters using the default resolution of 0.8, as well as resolution set to 1 to obtain a larger number of communities. Top 75 significant principal components, as selected with the JackStraw method, were used to compute uniform manifold approximation and projection (UMAP). To annotate individual cells with main cell types, we applied the SingleR package V.0.22^17^ using ImmGen gene expression compendium^18^ as the reference data set. This resulted in the most likely cell type being inferred for each single cell independently, in an unsupervised manner, based on the expression profile of the cell. Annotation significance p values were computed using SingleR built-in χ^2^ outlier test for the top-scored cell-type match. The resulting cellular origins were used to calculate cell-type proportions in BM and compare them between met-high and met-low mice. For determining proportion difference significance, the bias-corrected and accelerated (BCa) bootstrap method for independent two-samples was applied, together with one-sided hypothesis testing, using wBoot R Package V.1.0.3. Differential gene expression analysis and gene set enrichment analysis (GSEA) were performed using MAST,^19^ a package suited to analysis of sparse scRNA-seq, and clusterProfiler,^20^ respectively. GSEA reference signatures were obtained from MSIGDB^21^ (V.7.1—Hallmark (H), Regulatory Target Gene Sets (C3)).

### Cytokine array and ELISA

TCM was obtained from 5×10^6^ total tumor cells that underwent single-cell suspension and were subsequently cultured for 24 hours in serum-free medium. TCM from met-low or met-high B16 melanoma tumors was applied to a proteome profiler mouse XL cytokine array (ARY028; R&D systems) in accordance with the manufacturer’s instruction. The signals corresponding to each factor in the array were quantified by densitometry analysis. The ratio between the levels of the various factors in met-high and met-low TCM was calculated. IL-6 levels in TCM of met-low and met-high melanoma and breast cancers were quantified using a specific ELISA kit (ab46100; Abcam). The ELISA experiments were performed with five to eight mice per group and analyzed as mean±SD

### NanoString platform

For NanoString gene expression analysis, the gene panel nCounter PanCancer Progression was used (catalog no. XT-CSO-PROG1-12), which contains probes for 740 test genes and 30 housekeeping genes. RNA was purified with Single Cell RNA Purification kit (Norgen, Canada) from sorted CD45+ live cells obtained from met-low and met-high B16 melanoma, and analyzed with the nCounter platform. Data were filtered and normalized using a generalized linear model (R package, NanoStringDiff (V. 1.16.0))^22^—based on positive controls, housekeeping genes, and estimated background. Differential gene expression analysis was performed (met-high vs met-low) using either (1) a negative binomial model, akin to DESeq2^23^ and (2) area under the receiver operating characteristic (AUC-ROC) curves (R package, Seurat (V.3.1.4))^16^—collectively revealing seven significant differentially expressed genes (DEGs) (false discovery rate p<0.05, fold-change >1.25, AUC=1). An AUC value of 1 denotes the ability of a given gene to perfectly distinguish met-high from met-low.

### Multiomics data analysis for characterization of the cross talk between tumor cells and BM

To identify the axis responsible for the cross talk between tumor cells and the BM compartment, differential expression of cytokine receptor genes in the scRNA-seq BM data was analyzed and integrated with the tumor cytokine array findings. First, following SingleR cell-type annotation of individual cells, the Wilcoxon rank-sum test method using the Seurat R package was applied to identify, per cell type, the genes differentially expressed in met-high versus met-low groups. The testing was limited to genes showing, on average, at least 1.2-fold difference and those detected in at least 10% of cells in either of the groups. Multiple comparison adjustment was carried out using Benjamini-Hochberg correction. Next, cytokines in the array were paired to their respective receptors within the differentially expressed genes, based on the KEGG cytokine–cytokine receptor interaction pathway entry.^24^ This resulted in the list of cytokine–receptor pairs, per cell type, ranked by their cytokine expression ratio between the groups, receptor fold-change, and its adjusted p value of differential expression.

### Statistical analysis for in vitro and in vivo studies

Data are expressed as mean±SD. For in vitro studies, to ensure adequate statistical power, all experiments were performed with two technical repeats and at least three biological repeats. In the in vivo studies, mice that demonstrated pathological symptoms not related to their condition or disease were excluded from the study and the analysis. Mice were randomized before tumor implantation. The analysis of the results was performed blindly. At least five mice per group were used to reach statistical power considering a Gaussian distribution. The statistical significance of differences was assessed by one-way analysis of variance (ANOVA), followed by Turkey post hoc statistical test when comparing between more than two sets of data or by two-tailed Student’s t-test when comparing between two sets of data, using GraphPad Prism V.5 software (La Jolla, CA, USA). Significance was set at values of p<0.05.

## Results

### Metastatic tumors promote myeloid skewing through MDPs

To study the communication between the BM niche and metastatic tumor cells, we used tumor cell line pairs comprising one line that rarely metastasizes (met-low) and another line that metastasizes with high frequency (met-high). Cell lines within each pair originated from the same parental cell line, allowing a biologically relevant comparison. Mice were either subdermally implanted in the flank with met-low B16-F1 or met-high B16-F10 melanoma cells, or implanted in the mammary fat pad with met-low 67NR or met-high 4T1 breast cancer cells. In both tumor models, met-low and met-high primary tumors grew at similar rates, whereas lung metastasis was significantly increased in the met-high groups (online supplemental figure S1), in agreement with previous studies.^11 12^

When focusing on BM niche in met-high and met-low tumors, we observed increased percentage of LSK cells in the BM of mice implanted with tumors compared with tumor-free groups in both melanoma and breast cancer models (online supplemental figure S2A, B). Notably, met-high tumors exhibited the highest percentage of LSK cells in the BM niche of B16 melanoma, but such differences did not reach statistical significance in the breast cancer model. In addition, we noticed that LSK levels were substantially lower in the breast cancer model than in the melanoma model, probably due to known technical difficulty of Sca1+ immunostaining in BALB/c mice.^25^ In addition, the analysis of differentiated cells in the BM revealed a significant bias toward myeloid cells, compared with lymphoid cells in mice implanted with met-high tumors (online supplemental figure S2C, D). Moreover, mice implanted with met-high tumors exhibited the highest number of CFUs derived from HSPCs in peripheral blood, further indicating increased hematopoiesis (online supplemental figure S2E). Taken together, metastatic tumors promote myelopoiesis.

**Figure 1:**
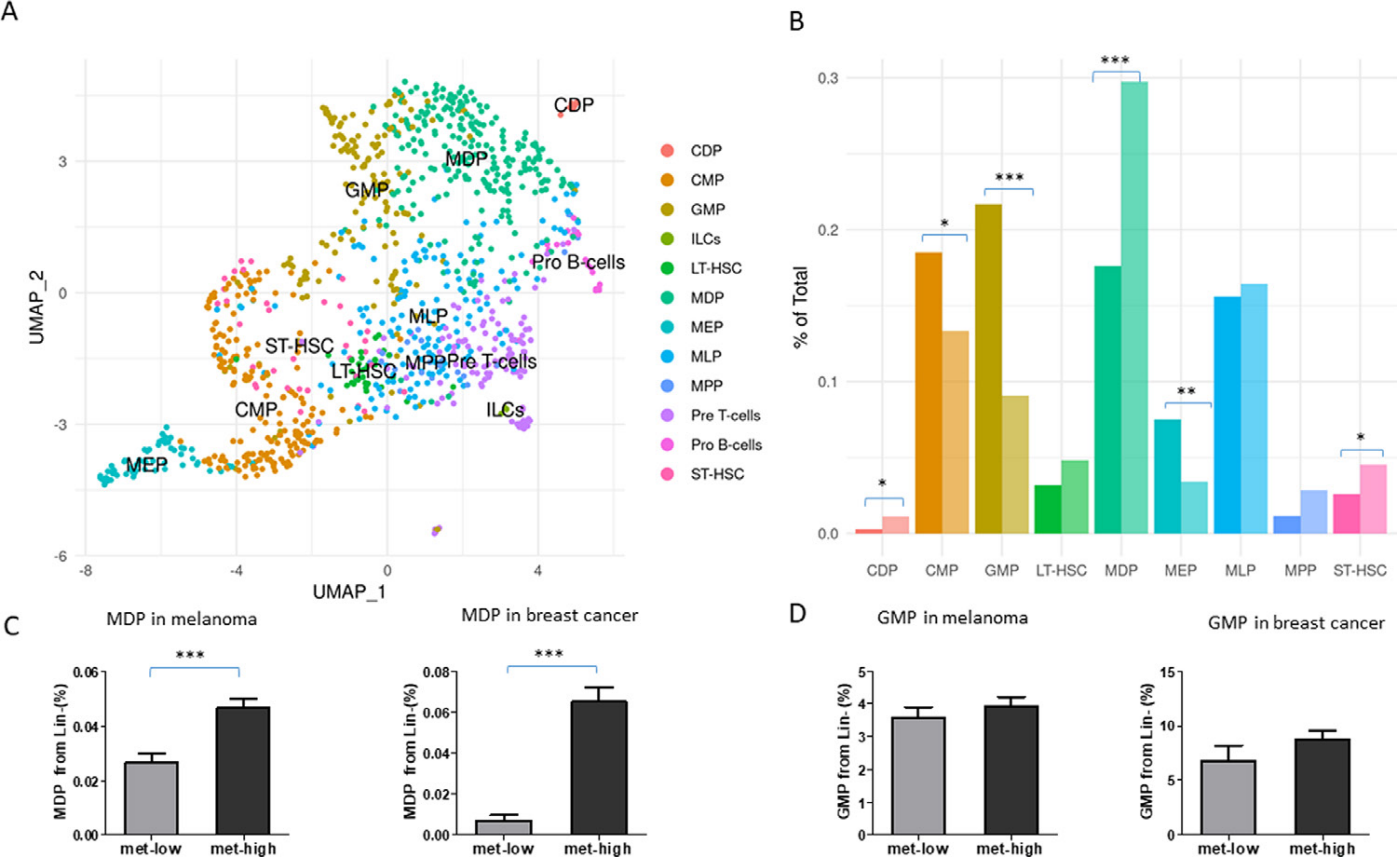
Metastatic tumors promote myeloid skewing through MDPs. Mice were subdermally implanted in the flank with met-low B16-F1 or met-high B16-F10 melanoma cells (5×10^5^ cell/mouse). At end point, LSK cells were sorted from the bone marrow and analyzed by single-cell RNA sequencing. (A) UMAP plots of LSK clustering and individual cell-type annotation by SingleR for both groups, using ImmGen gene expression compendium as a reference. MDP and GMP clusters are indicated by blue and red arrows, respectively. (B) Stem and progenitor enrichment levels in met-low and met-high groups, calculated by BCa bootstrap independent two-samples test for proportion differences. (C–D) In a separate experiment, mice were either subdermally implanted in the flank with met-low B16-F1 or met-high B16-F10 melanoma cells (5×10^5^ cell/mouse), or implanted in the mammary fat pad with met-low 67NR or met-high 4T1 breast cancer cells (5×10^5^ cell/mouse). At end point (day 18 and day 21, respectively), mice were sacrificed and bone marrow was obtained. Flow cytometry validation of MDP (C) and GMP (D) percentage from Lin− cells of met-low and met-high tumor-bearing mice was performed. Melanoma and breast cancer models are shown (n=5 mice/group in melanoma model, n=5–10 mice/group in breast cancer model). Statistical significance was assessed using unpaired two-tailed t-test and shown as *p<0.05; **p<0.01; and ***p<0.001. BCa, bias-corrected and accelerated; CDP, common dendritic progenitor, CMP, common myeloid progenitor, GMP, granulocyte-monocyte progenitor; LT-HSC, long term hematopoietic stem cell; MDP, myeloid dendritic progenitor; MEP, megakaryocyte erythroid progenitor; MLP, multilymphoid progenitor; MPP, multipotent progenitor; ST-HSC, short term hematopoietic stem cells. LSK, Lin-Sca1+Kit+; Mmet-high, high metastatic potential; met-low, low metastatic potential; UMAP, uniform manifold approximation and projection.

To address our hypothesis of early HSPC programming by highly metastatic cells, we performed scRNA-seq of LSK cells obtained from the BM of mice implanted with met-low or met-high B16 melanoma. Naïve tumor-free mice served as a control group. We identified the specific cellular composition of the LSK cells by correlating single-cell expression profiles with ImmGen-sorted cell reference transcriptomic database (figure 1A).^17 18^ Importantly, among the LSK differentiation pattern-enriched cells, MDPs were most significantly and highly enriched in met-high tumor group, whereas GMPs were most significantly and highly enriched in met-low tumor group (p<0.0001, for both MDP and GMP, BCa bootstrap independent two-samples test for proportion differences) (figure 1B). Furthermore, we found that CX3CR1 is enriched within the MDP cells of the met-high tumor group (online supplemental figure S3A, B, p=0.041, Wilcoxon rank-sum test), in line with a previous study demonstrating that MDPs are initially identified as CX3CR1+ cells.^26^ In addition, the transcription factor IRF2BP2, which has been reported to regulate macrophage activity,^27^ was also upregulated in MDPs from met-high tumors compared with MDPs from met-low tumors. To validate the scRNA-seq results, we quantified the percentage of MDPs, GMPs, and other altered progenitors in BM of met-low and met-high tumor-bearing mice. MDPs were significantly elevated in met-high melanoma and breast cancer compared with met-low cancers, whereas all other annotated cells including GMPs were not significantly changed (figure 1C, D and online supplemental figure S3C–F).

Next, to further study the functional differences of MDPs in met-high and met-low tumors, we analyzed DEGs in the MDP population in the BM of tumor-free, met-low, and met-high melanoma- bearing mice. Genes differentially expressed in naïve and met-low MDPs (vs met-high MDPs) significantly overlap (n=62; online supplemental figure S3G). They are involved in similar immune-related pathways (online supplemental figure S3H) and are potentially controlled by identical transcription factors (online supplemental figure S3I). Notably, the serum response factor (SRF) network—known to be involved in myeloid-dendritic differentiation^28^—is enriched in naïve and met-low MDPs. Loss of SRF results in accumulation of undifferentiated hematopoietic stem cells^28^— consistent with increased LSK levels within the BM of met-high tumor-bearing mice. In contrast, met-high MDPs are enriched for E2F-target genes involved in mitosis and the G2M checkpoint, suggesting that they possess higher proliferative potential. Overall, these results suggest that met-low MDPs remain ‘naïve-like’ and that met-high tumors specifically educate HSPCs by enriching the MDP population and altering its differentiation program.

### Metastatic tumors dictate a long-lived education of HSPCs into myeloid-biased differentiation

**Figure 2:**
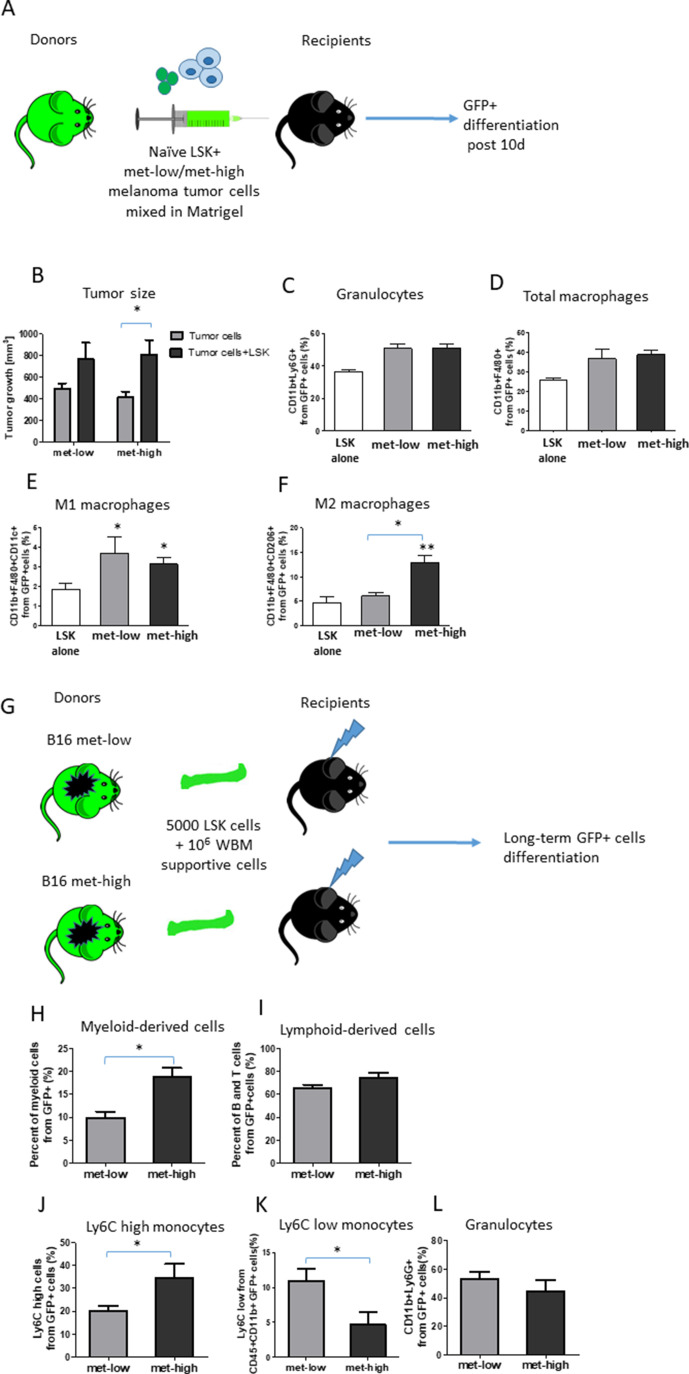
Metastatic tumors dictate a long-lived education of hematopoietic stem and progenitor cell differentiation toward myelopoiesis. (A) A schematic representation of the LSK and tumor cell co-implantation experiment is shown. LSK cells obtained from naïve GFP-expressing donor mice were coinjected with either met-low or met-high melanoma tumor cells to recipient mice. Control recipient mice (not shown) were injected either with LSK cells alone or with tumor cells alone. At end point, mice were sacrificed and tumors harvested. GFP+ progeny in tumors was assessed by flow cytometry (n=4–5 mice/group). (B) Tumor volumes at end point are presented. (C–F) The percentages of granulocytes (C), total macrophages (D), M1 macrophages (E), and M2 macrophages (F) in tumors were determined by flow cytometry on GFP+ gated cells. (G) A schematic representation of the tumor-educated LSK transplantation experiment is shown. LSK cells were obtained from the bone marrow of donor mice harboring met-low or met-high melanomas. The LSK cells (5×10^3^) were injected together with whole bone marrow supportive cells (1×10^6^) from naïve mice into lethally irradiated naïve recipient mice. Donor engraftment was monitored for 16 weeks after transplantation. GFP+ progeny in peripheral blood was assessed by flow cytometry. (H–I) Lineage distribution of myeloid-derived (H) and lymphoid-derived cells (I) is shown. (J–L) The percentage of GFP+ Ly6C^high^ monocytes (J), Ly6C^low^ monocytes (K), and GFP+ Ly6G+granulocytes (L) was gated from myeloid CD11b+ cells (n=5 mice/group). Statistical significance was assessed by one-way analysis of varaince, followed by Tukey post hoc test when comparing more than two groups or unpaired two-tailed t-test when comparing two groups. Asterisks represent significance from control, unless indicated otherwise in the figure. Significant p values are shown as *p<0.05; **p<0.01; ***p<0.001. LSK, Lin-Sca1+Kit+; met-high, high metastatic potential; met-low, low metastatic potential; WBM, whole bone marrow.

To further study the changes in myeloid-biased HSPC differentiation in met-high compared with met-low tumors, LSK cells obtained from naïve GFP-expressing mice were mixed with either met-low or met-high melanoma tumor cells in Matrigel and subsequently implanted in recipient mice (figure 2A). Control recipient mice were implanted with Matrigel containing either LSK cells or tumor cells (met-low or met-high cells). After 10 days, tumor size and differentiation of GFP+ cells in tumors, peripheral blood, and BM were assessed. Tumor size was significantly increased in mice implanted with the mixture of HSPCs and met-high tumor cells, an effect which was not statistically significant in the corresponding met-low tumor group (figure 2B). These results suggest that HSPCs give rise to hematopoietic cell subsets that support tumor growth. When comparing between met-high and met-low tumors in the context of GFP+ HSPC differentiation, no significant differences were detected in the percentages of GFP+ granulocytes, total macrophages, and proinflammatory M1 macrophages. However, the level of tumor-supporting GFP+ M2 macrophages was significantly increased by approximately two-fold in met-high tumors (figure 2C–F). No GFP+ cells were detected in peripheral blood or BM (data not shown). Taken together, our results demonstrate that in met-high tumors, HSPCs undergo myeloid-biased differentiation, an effect known to promote tumor aggressiveness and metastasis.

Next, to study the long-lived education and differentiation of HSPC by tumor cells, LSK cells obtained from BM of GFP-expressing mice harboring either met-low or met-high melanoma tumors were transplanted along with whole BM (GFP-supporting) cells into lethally irradiated naïve recipient mice (figure 2G). After 16 weeks, the percentages of circulating GFP+ myeloid and lymphoid cells in the peripheral blood were evaluated. A significant increase in the percentage of myeloid but not lymphoid cells was found in mice transplanted with BM cells originating from met-high donors (figure 2H–I). Moreover, in the met-high group, the proinflammatory Ly6C^high^ monocyte fraction was significantly increased in circulating tested GFP+ myeloid cells, while there was a decrease in Ly6C^low^ monocytes that exhibit a low inflammatory profile (figure 2J–K). No difference was observed in the granulocyte population (figure 2L). These results indicate that met-high tumors dictate a long-lived direct differentiation of HSPC toward myeloid cells, particularly differentiating into M2 macrophages in tumors and proinflammatory monocytes in peripheral blood.

To further strengthen the correlation between HSPC differentiation and immune cell composition in the tumor microenvironment of met-high and met-low tumors, we used high-throughput mass cytometry (CyTOF) to analyze the percentage of different myeloid-derived cells in the stroma of met-high or met-low tumors followed by flow cytometry validation of specific cell types for both melanoma and breast cancer. Overall, the analysis of both CyTOF and flow cytometry data (online supplemental figure S4) revealed a significant enrichment in M2 macrophages in met-high tumors compared with met-low tumors, whereas the levels of proinflammatory M1 macrophages were significantly decreased. In addition, the levels of Ly6C^high^ monocytes were increased in met-high tumors in comparison with met-low tumors, while dendritic cells were decreased (online supplemental figure S4A). Of note, the granulocyte population was also significantly increased in met-high tumors in the melanoma but not breast cancer model (online supplemental figure S4B, C). Overall, these results demonstrate similar trends to those found in the LSK BM transplantation experiment, indicating that met-high tumors dictate a specific differentiation of HSPCs toward myeloid cells, especially M2 macrophages.

### IL-6 promotes MDP proliferation and differentiation

**Figure 3:**
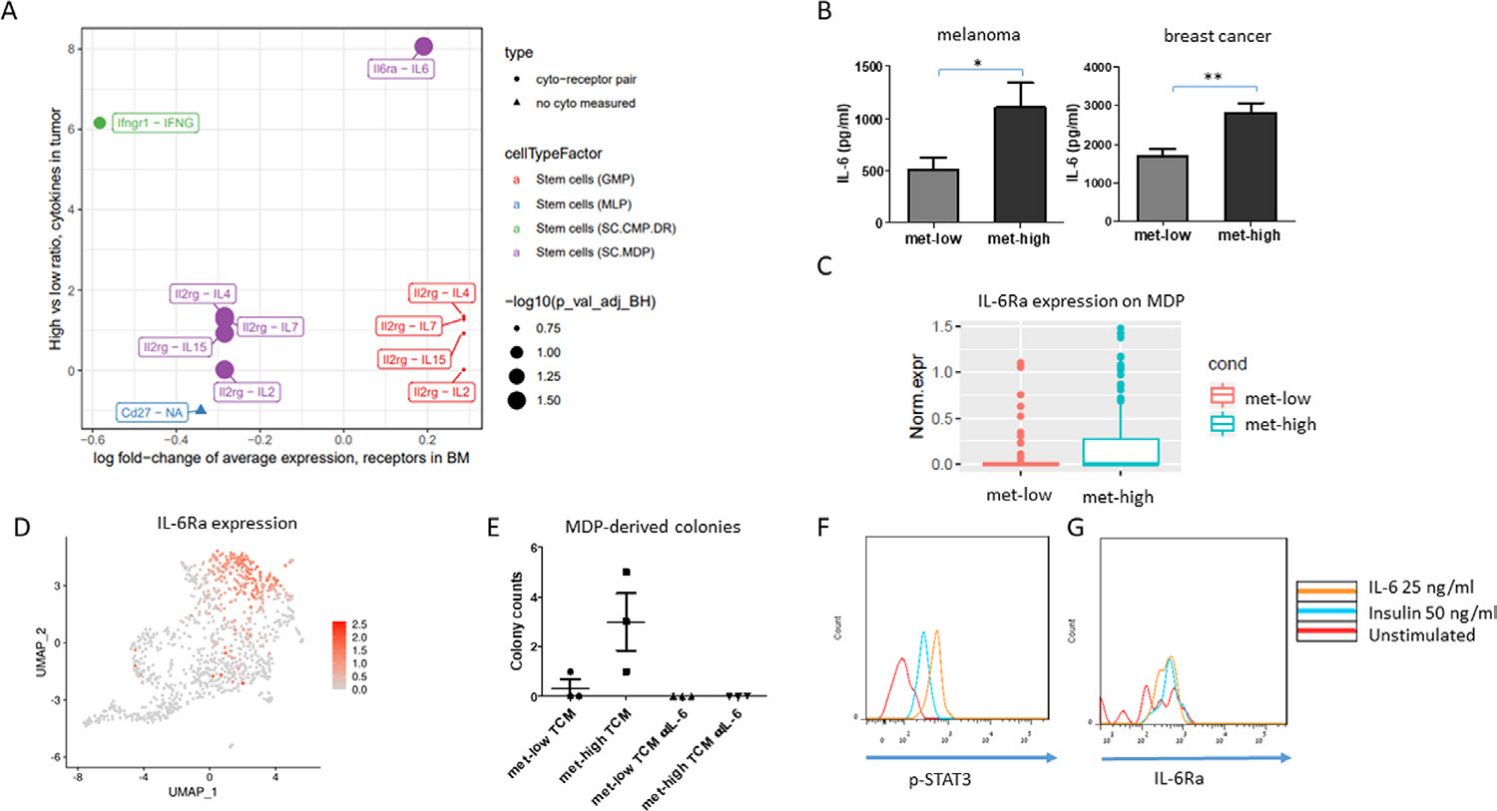
IL-6 is a major mediator of the cross talk between tumors and MDPs. (A) Mapping upregulated cytokines in melanoma met-high TCM with their corresponding receptors, differentially expressed by progenitor populations, as identified by single-cell RNA sequencing (Wilcoxon rank-sum test followed by Benjamini-Hochberg correction). (B) IL-6 levels were quantified by ELISA in met-low and met-high TCM of melanoma and breast cancer models (n=5–8= mice/group). (C) IL-6Ra expression in the MDP population in the bone marrow of met-low and met-high melanoma-bearing mice, based on scRNA-seq data set (average fold-change=1.21, p=0.02, Wilcoxon rank-sum test). (D) Single-cell IL-6Ra messenger RNA expression labeling on the UMAP plot. (E) MDPs from naïve mice were grown in Methocult medium supplemented with met-low or met-high melanoma TCM in the presence or absence of anti-IL-6 neutralizing antibodies. MDP-derived colonies were counted (n=3 biological repeats/group). (F–G) MDPs obtained from the bone marrow of naïve mice were stimulated with escalating doses of IL-6. The levels of p-STAT3 (F) and IL-6Ra (G) were determined by flow cytometry. Statistical significance was assessed by one-way analysis of varaince, followed by Tukey post hoc test when comparing more than two groups or unpaired two-tailed t-test when comparing two groups. Asterisks represent significance from control, unless indicated otherwise in the figure. Significant p values are shown as *p<0.05; **p<0.01. BM, bone marrow; GMP, granulocyte-monocyte progenitor; IL-6, interleukin 6; IL-6Ra, interleukin 6 receptor; MDP, monocyte-dendritic progenitor; met-high, high metastatic potential; met-low, low metastatic potential; MLP, multilymphoid progenitor; SC.CMP.DR, stem cell - common myeloid progenitor; TCM, tumor-conditioned medium; UMAP, uniform manifold approximation and projection.

We hypothesized that the cross talk between tumor and BM cells requires messenger proteins, for example, cytokines. With the focus on MDPs, we searched for DEGs in MDPs between met-high and met-low groups. Overall, 207 genes were significantly altered between the two groups as shown in the volcano plot, including receptors and transmembrane proteins, such as IL-6 receptor (IL-6Ra) and interelukin 2 receptor (online supplemental figure S5A and online supplemental table S1). We next sought to identify tumor-secreted factors that correlate with the scRNA-seq data. A cytokine array to quantify the levels of a broad range of factors in the TCM of met-high and met-low melanomas was used. Several factors, including CXCL1, CXCL10, interleukin 3, IL-6, and interleukin 13, were found at higher levels in the TCM of met-high tumors (online supplemental figure S5B). We then matched the cytokines to their corresponding receptors mentioned above. Using this approach, IL-6 and its receptor were found to be good candidates for mediating the cross talk between tumor cells and HSPCs (figure 3A). Verification by ELISA revealed that IL-6 was significantly upregulated in met-high TCM in both melanoma and breast cancer models (figure 3B). IL-6Ra was found to be upregulated in MDPs of mice harboring met-high melanoma (figure 3C; average fold-change=1.21, p=0.02, Wilcoxon rank-sum test), and its expression is abundant in the MDP population shown in the UMAP plot (figure 3D). These results were verified by flow cytometry for both melanoma and breast cancer models (online supplemental figure S5C). Furthermore, to strengthen the involvement of IL-6 in met-high tumors, transcription signatures of CD45+ stroma cells obtained from the tumor microenvironment of B16 melanoma, assessed by NanoString approach, revealed seven upregulated genes, including THBS1, MAPKAPK2, CD9, TXNIP, CD24, ITGA5, and SYK (online supplemental figure S5D). Importantly, some of these genes (THBS1, MAPKAPK2, CD9, TXNIP) were strongly associated with IL-6 signaling,^29–31^ indicating that IL-6 or its associated genes are coupled with metastatic tumors, in alignment with our previous findings. Overall, these findings may explain the increased myeloid bias in met-high compared with met-low tumors.

10.1136/jitc-2021-002856.supp1 Supplementary data

To directly assess the effect of IL-6 on MDP proliferation and differentiation, MDP cells isolated from BM of naïve mice were seeded in Methocult medium supplemented with met-high or met-low TCM in the presence or absence of neutralizing IL-6 antibody. Following 12 days of incubation, myeloid colonies were scored. MDP cells formed more monocyte colonies in the presence of met-high TCM in comparison with the met-low group. Importantly, neutralizing IL-6 in the met-high group completely abolished MDP growth and colony formation (figure 3E). However, naïve GMPs cultured with met-low and met-high TCM in the presence or absence of IL-6 resulted in few colonies with no significant difference between all groups (online supplemental figure S5E), suggesting that the major effect of IL-6 in HSPCs is on MDP population.

One of the downstream effects in the IL-6 pathway is phosphorylation of STAT3.^32^ To verify that MDPs respond directly to IL-6, we stimulated BM cells obtained from naïve mice with IL-6 for 30 min and evaluated p-STAT3 expression specifically in the MDP population. Indeed, p-STAT3 levels in MDP cells were increased on stimulation, with IL-6 demonstrating that IL-6 directly affects IL-6Ra-expressing MDPs (figure 3F, G). Taken together, our results suggest that IL-6 secreted by highly metastatic tumors directly promotes myeloid-biased HSPC differentiation into MDPs but not GMPs.

### IL-6-dependent MDP-derived macrophages promote metastasis

**Figure 4:**
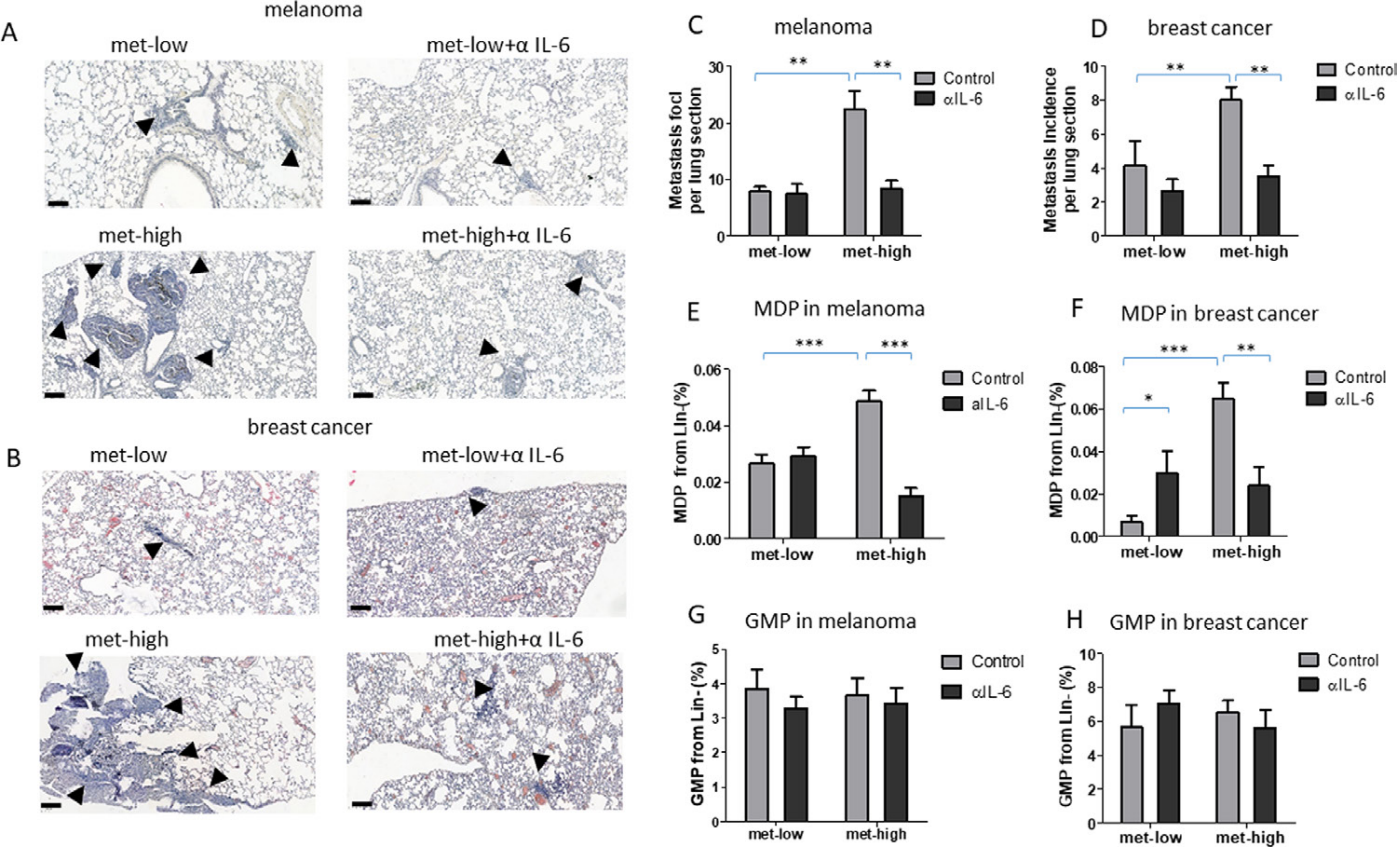
IL-6 pathway blockade inhibits metastasis. Mice were implanted with met-low or met-high melanoma or breast cancer cells. One week later, mice were treated with IgG (control) or anti-IL-6 antibodies twice weekly. At end point, mice were sacrificed, lungs were removed, and bone marrow was harvested (n=5–8 mice/group). (A–B) Representative images of lung sections from melanoma (A) and breast cancer (B) are shown, bar=100 µm. Arrows indicate metastatic foci. (C–D) Metastatic foci per lung section were quantified (n=5–7 sections/mouse) for melanoma (C) and breast cancer (D). (E–F) MDP levels in BM of melanoma (E) and breast cancer (F) were assessed by flow cytometry. (G–H) GMP levels in BM of melanoma (G) and breast cancer (H) were assessed by flow cytometry. Statistical significance was assessed by one-way analysis of variance, followed by Tukey post hoc test when comparing more than two groups or unpaired two-tailed t-test when comparing two groups. Asterisks represent significance from control, unless indicated otherwise in the figure. Significant p values are shown as *p<0.05; **p<0.01; ***p<0.001. BM, bone marrow; GMP, granulocyte-monocyte progenitor; IL-6, interleukin 6; MDP, monocyte-dendritic progenitor; met-high, high metastatic potential; met-low, low metastatic potential.

We next sought to investigate the effect of IL-6 signaling in vivo focusing on primary tumor growth, metastasis and HSPC differentiation. To this end, mice were implanted with met-low or met-high melanoma or breast cancer cells, and subsequently treated with either IgG control or anti-IL-6 antibodies. The rates of primary tumor growth were similar in all groups throughout the course of the experiment (online supplemental figure S6A, B). However, in both tumor models, anti-IL-6 treatment dramatically reduced the incidence of lung metastasis only in mice harboring met-high tumors (figure 4A–D). This effect was accompanied by a significant decrease in MDP levels in the BM of these mice, whereas GMP levels did not significantly change in any of the groups tested (figure 4E, F, G, H). These results suggest that tumor-educated MDPs but not GMPs correlate with IL-6-mediated metastasis in met-high tumors.

**Figure 5:**
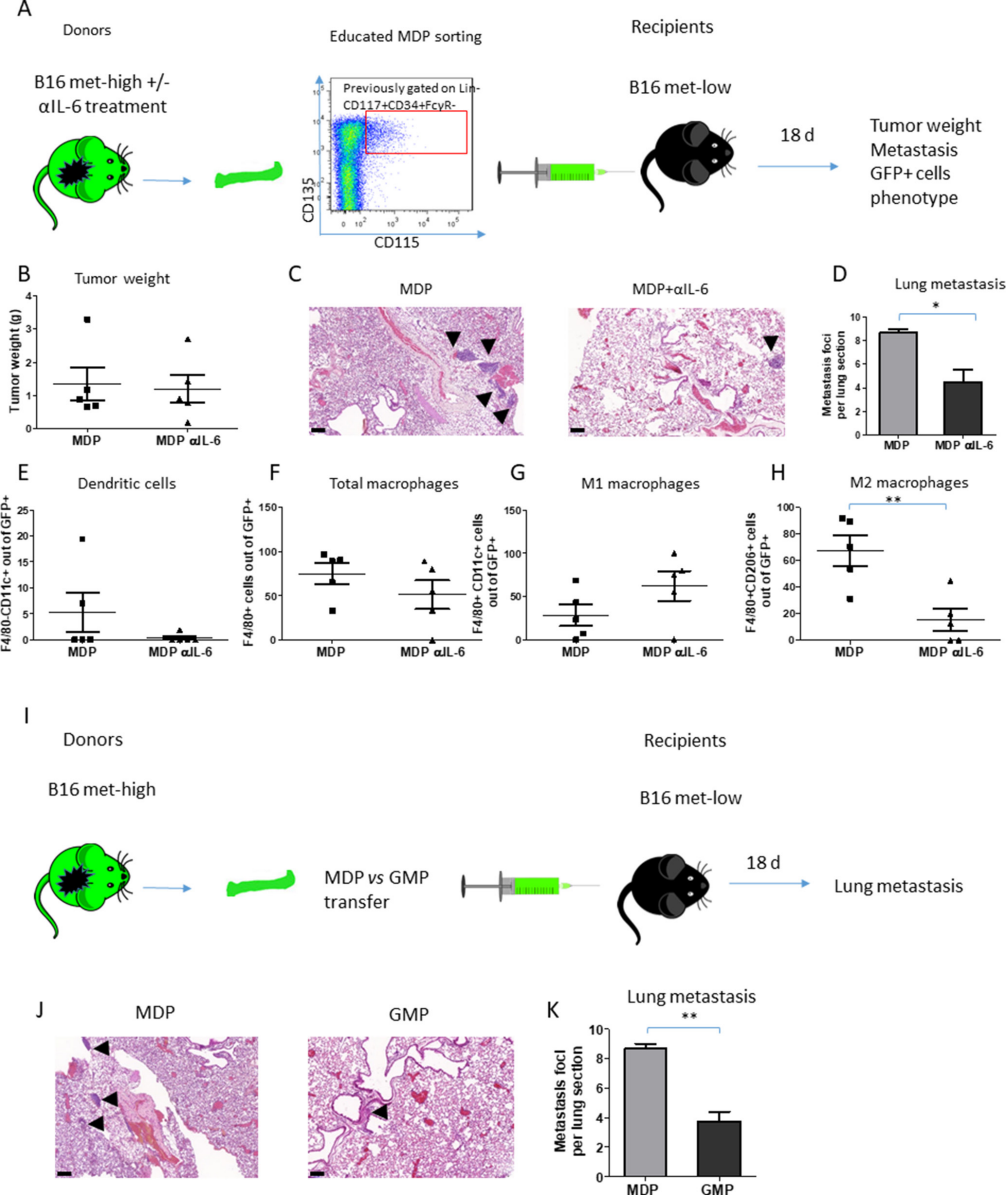
MDP-induced metastasis through M2 macrophages is dependent on IL-6 pathway. (A) A schematic representation of MDP adoptive transfer experimental design is shown. GFP-expressing mice implanted with met-high melanoma cells were treated with either IgG control or anti-IL-6 antibodies twice weekly. At end point, FACS-sorted MDPs were intravenously injected into naïve recipient mice (n=5 mice/group). The next day, the recipient mice were implanted with met-low melanoma cells. At end point, mice were sacrificed, and tumors and lungs were extracted. (B) Tumor weights are shown. (C) Representative images of lung sections are shown, bar=100 µm. Arrows indicate metastatic foci. (D) Metastatic foci per lung section were quantified (n=5–7 sections per group). (E–H) GFP+ cells in single-cell suspensions of lung samples were evaluated by flow cytometry to quantify the percentage of dendritic cells (E), total macrophages (F), M1 macrophages (G), and M2 macrophages (H). (I) A schematic representation of MDP versus GMP adoptive transfer is shown. GFP-expressing mice were implanted with met-high melanoma cells. At end point, MDPs or GMPs were sorted by FACS and were subsequently intravenously injected into naïve recipient mice (n=4 mice/group). The next day, the recipient mice were implanted with met-low melanoma cells. At end point, mice were sacrificed, and lungs were removed for the evaluation of metastasis. (J) Representative images of lung sections are shown, bar=100 µm. Arrows indicate metastatic foci. (K) Metastatic foci per lung section were quantified (n=3–4 sections/mouse). Statistical significance was assessed by unpaired two-tailed t-test. Significant p values are shown as *p<0.05; **p<0.01; ***p<0.001. GMP, granulocyte-monocyte progenitor; IL-6, interleukin 6; MDP, monocyte-dendritic progenitor; met-high, high metastatic potential; met-low, low metastatic potential.

We next directly tested the effect of tumor-educated MDPs on metastasis in vivo by performing an MDP adoptive transfer experiment. To this end, GFP-expressing donor mice were implanted with met-high melanoma cells and then treated with control IgG or anti-IL-6 antibodies. GFP+ MDPs from these mice were then adoptively transferred into recipient mice subsequently injected with met-low tumor cells, as schematically illustrated in figure 5A. No significant difference in tumor growth was observed between the two groups (figure 5B). The number of metastatic lesions in the lungs of mice adoptively transferred with MDPs obtained from anti-IL-6-treated mice was significantly reduced in comparison with MDP from IgG-treated control mice (figure 5C, D).

As MDPs give rise to monocytes and DCs,^33^ we next evaluated these two populations in lung single-cell suspensions of mice adoptively transferred with MDPs obtained from mice treated with anti-IL6 or IgG control. DCs were barely detected in the GFP+ anti-IL-6 treated MDP cell population (figure 5E). Furthermore, there were no differences in the levels of GFP+ total macrophages and M1 macrophages between the two groups (figure 5F, G). However, the percentage of M2 GFP+ macrophages was significantly decreased in the lungs of mice adoptively transferred with MDPs obtained from anti-IL-6-treated mice, indicating that IL-6 blockade induces a functional change in MDPs (figure 5H). Of note, these results are in line with the CyTOF findings demonstrating that DC levels are reduced in met-high tumors compared with met-low tumors (online supplemental figure S4A).

Next, as GMPs can also differentiate into monocytes,^10^ in a separate experiment met-high-educated MDPs versus GMPs were adoptively transferred into mice bearing met-low tumors, as illustrated in figure 5I, and lung metastasis was evaluated. A significant decrease in the number of pulmonary metastasis was observed in the mice adoptively transferred with GMP compared with MDPs (figure 5J, K), suggesting that MDPs but not GMPs contribute to metastasis. Overall, these findings suggest that MDPs rather that GMPs, upon IL-6 secretion from highly metastatic tumors, undergo a direct specific differentiation pattern toward M2 macrophages but not DCs at the metastatic sites and promote metastasis.

### IL-6-induced MDP differentiation is a driver protein of the metastatic switch

**Figure 6:**
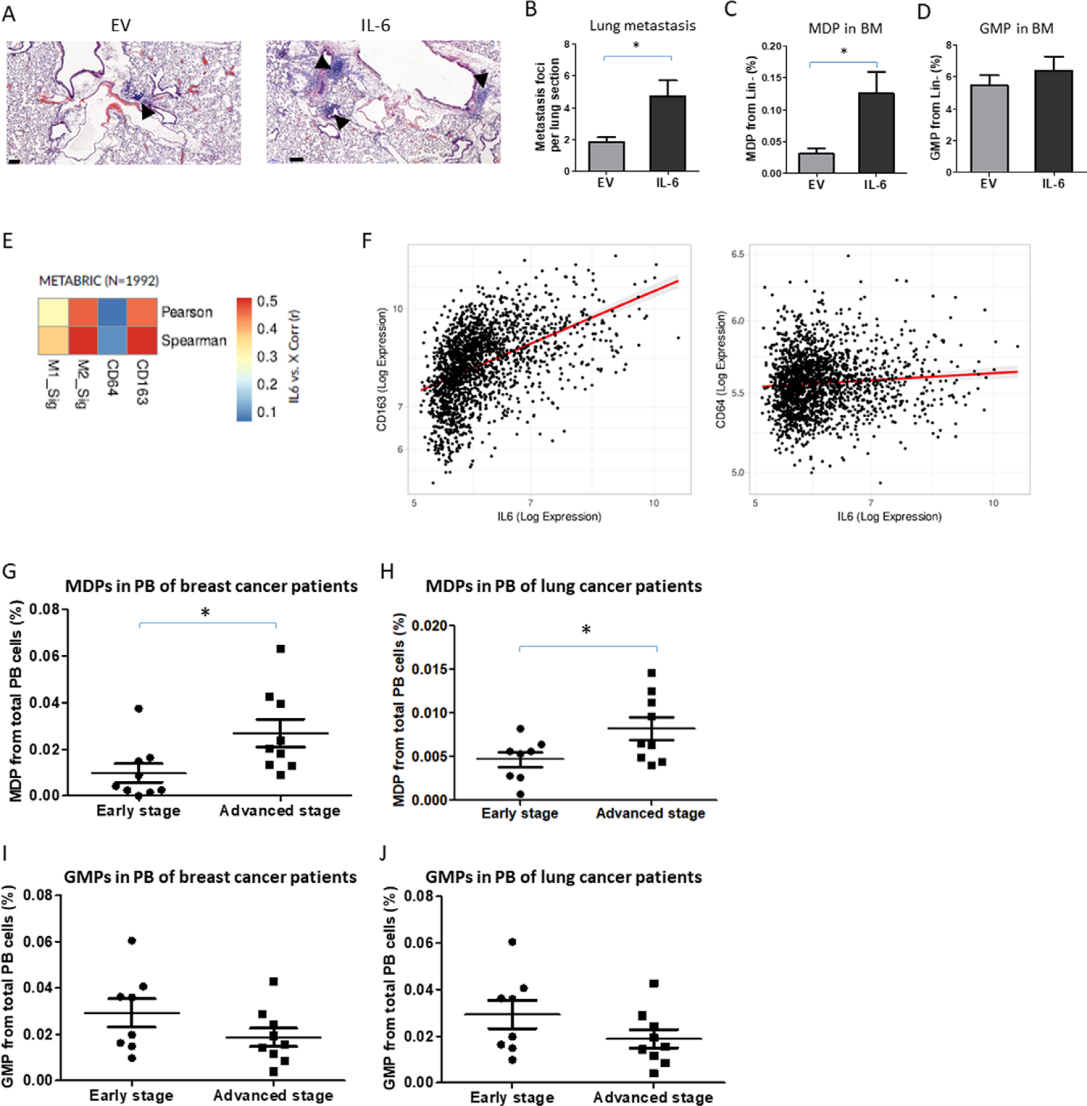
Increased immunosuppressive macrophages and MDPs correlate with IL-6 expression and aggressive tumors. (A–D) C57BL/6 mice aged 8–10 weeks were implanted with B16 IL-6 overexpressing cells or with corresponding control EV cells. At end point (day 18), mice were sacrificed, lungs were removed, and the bone marrow was harvested (n=4–6 mice/group). (A) Representative images of lung sections are shown, bar=100 µm. Arrows indicate metastatic foci. Metastatic foci per lung section were quantified (n=4–6 sections/mouse). (C) MDP and (D) GMP levels in BM were assessed by flow cytometry. (E) Heat-map of linear correlations in R values between IL-6 expression and M1 or M2 gene signatures as well as single genes CD64 or CD163 representing M1 and M2 subsets, respectively. The data were obtained from human breast cancer samples using the METABRIC data set. (F) Scatter graphs of IL-6 expression in correlation with CD64 or CD163 in human breast cancer samples (METABRIC data set). (G–J) MDP (G, H) and GMP (I, J) levels were analyzed in peripheral blood of patients with breast (G, I) and lung (H, J) cancer segregated based on early and advanced stage disease. Statistical significance was assessed by unpaired two-tailed t-test. Significant p values are shown as *p<0.05. BM, bone marrow; EV, empty vector; GMP, granulocyte-monocyte progenitor; IL-6, interleukin 6; MDP, monocyte-dendritic progenitor; PB, peripheral blood.

To demonstrate that IL-6 is responsible for a metastatic switch in our experimental settings, we generated met-low melanoma overexpressing IL-6 or EV control (B16-F1-IL-6 and B16-F1-EV, respectively). IL-6 expression in cultured cells was verified by ELISA (online supplemental figure S7A), and the two cell lines exhibited similar proliferation rates (online supplemental figure S7B). Next, mice were implanted with met-low B16-F1-IL-6 or B16-F1-EV and tumor growth was assessed. There was no significant difference in tumor growth between the two groups (online supplemental figure S7C), while a significant increase in IL-6 was found in the TCM of B16-F1-IL-6 group compared with B16-F1-EV (online supplemental figure S7D), suggesting that IL-6 has no direct protumorigenic role in the primary tumor microenvironment. However, the incidence of lung metastasis was significantly increased in B16-F1-IL-6 tumor-bearing mice (figure 6A, B). Moreover, the elevation in lung metastasis was directly associated with increased MDP levels detected in the BM (figure 6C), whereas the levels of GMPs, common myeloid progenitors (CMPs), and megakaryocyte-erythroid progenitors (MEPs) remained unchanged between the two groups (figure 6D and online supplemental figure S7E, F). These results further indicate that IL-6 contributes to the metastatic switch mediated in part by HSPC differentiation into MDPs that further differentiate into M2 macrophage, supporting metastasis.

To further correlate between MDP and macrophages supporting metastasis in human samples, we analyzed bulk RNA-seq from patients with breast carcinoma using the METABRIC data set.^34^ A moderate, positive correlation (r=~0.4–0.5) was found between IL-6 expression and the molecular signature of M2 macrophages (CD163, CD204, CD206, CD200R1, TGM2, IL1R2), whereas little correlation was found with an M1 gene signature (CD64, MHCII, CD86, CD80, CD68, NOS2) (figure 6E). This trend is strengthened when considering a single marker gene largely unique to each macrophage subtype (figure 6F), suggesting that a similar mechanism may exist in humans.

To confirm the correlation of circulating MDPs with tumor aggressiveness, we analyzed peripheral blood samples from patients with breast and lung cancer, segregated into early or advanced cancer stages. Consistent with the murine data, MDP levels were significantly higher in patients with aggressive tumors, whereas GMP levels did not significantly change between the groups (figure 6G–J). These results further indicate a new role for MDPs in promoting tumor aggressiveness and likely suggest that MDPs may serve as a potential biomarker to monitor metastatic potential in patients with cancer. Overall, these findings suggest that IL-6 directly affects MDPs, which in turn, differentiate into metastasis-promoting cells, thereby promoting tumor aggressiveness, an effect which may also exist in humans.

## Discussion

Our study is the first to compare the BM niche education in high metastatic versus low metastatic tumors. We demonstrate that high metastatic tumors derive a unique programming of HSPCs, specifically to MDP population. This effect is mediated by IL-6 signaling, which further differentiates MDPs into Ly6C^high^ monocytes but not DCs. These monocytes give rise to M2 macrophages that support metastasis. Consistent with the murine data, our study also demonstrates that MDPs rather than GMPs are correlated with tumor aggressiveness in patients with cancer, suggesting a direct link between HSPC programming toward prometastatic myeloid cells and their outcome on metastasis.

Tumor progression is linked to pronounced perturbations in myelopoiesis, similar to inflammation, resulting in increased levels of committed and early myeloid progenitor populations.^6 7^ During tumor-induced myelopoiesis, HSPCs migrate from the BM and accumulate at distant organ sites. There, they differentiate into tumor-supporting myeloid cells to drive immunosurveillance,^35^ to participate in the formation of premetastatic niche,^2 36^ and to facilitate tumor cell seeding.^2^ A recent study has demonstrated that early progenitors can be found in the blood circulation of patients with different types of solid tumors, with higher levels of GMPs that eventually differentiate into granulocytes. Moreover, increased levels of myeloid progenitors correlated with overall poor outcome in these patients.^7^ In agreement, we observed myeloid bias in peripheral blood associated with highly metastatic tumors, as demonstrated by BM transplantation experiments using educated LSK cells obtained from met-low or met-high tumor-bearing mice. Using scRNA-seq of LSK cells obtained from mice harboring met-high tumors revealed a gene signature that reflects an MDP differentiation pattern. These cells were then found to serve as the origin of tumor-associated macrophages in the pulmonary metastatic sites. These findings strongly support the concept that met-high tumors affect the HSPC progeny to support metastatic-promoting accessory cells. Importantly, these effects were absent in met-low tumors, which resulted in gene signature that is closely related to naïve HSPCs. Moreover, the BM transplantation experiment using educated LSK cells from tumor-bearing mice further suggests that HSPC fate is long-lived as they maintain their biased progeny in BM of reconstituted mice over a 3-month period. In fact, sustained met-high-educated HSPC-specific differentiation suggests the involvement of epigenetic modifications that might regulate their programming, a process that should be further elucidated. Furthermore, while there is an overwhelming amount of evidence for HSPCs giving rise to MDSCs in tumor conditions,^8^ we found that LSK cells educated by met-low or met-high tumor conditions exhibit an MDP phenotype, with specific differentiation program which further gives rise to M2 macrophages only in met-high tumors. Indeed, studies have demonstrated that myeloid cells and tumor-associated immunosuppressive macrophages (M2 macrophages) home to the premetastatic sites where they contribute to the recruitment and retention of circulating tumor cells.^5 36 37^ In agreement with these studies, here we provide further evidence for the origin of M2 macrophages, because we demonstrate that highly metastatic tumors promote metastasis through enrichment of MDP-derived macrophages localized at the metastatic sites. We further demonstrate that these effects exist only in met-high tumors but not in met-low tumors, suggesting a direct involvement of tumor cells in HSPC progeny. The existence of two distinct and independently regulated cellular pathways challenges the function of GMP-derived versus MDP-derived monocytes and their relative contribution to metastasis. Here we suggest that MDPs but not GMPs have a novel function in the metastatic switch. Importantly, we demonstrate that, in the context of cancer, the differentiation pattern of MDPs is specific to monocytes and not DCs, as previously demonstrated,^9^ further indicating their education toward prometastatic cells that support the metastatic switch.

HSPCs express receptors of various inflammatory cytokines, such as interleukin 1, IL-6, interferon ʎ and Toll-like receptor (TLR) ligands, making them sensitive to external stimuli.^38–40^ It has been suggested that such receptors regulate HSPC differentiation. Thus, tumors may use this opportunity to condition the BM compartment via factors secreted by the tumor microenvironment. Our experimental model revealed an association between upregulated IL-6 levels in met-high TCM and elevated expression levels of IL-6Ra in met-high-educated MDPs. The IL-6/IL-6Ra pathway is often hyperactivated in many types of cancer^41^ and plays a key role in proliferation, survival, and invasiveness of tumor cells.^42 43^ In addition, it has been shown that IL-6 mediates metastatic mechanisms such as stimulation of epithelial-to-mesenchymal transition^44^ and induction of matrix metalloproteinases.^45^ Our study, however, provides another role for IL-6 in metastasis. We show that IL-6 induces metastasis through programming HSPCs toward tumor-supporting metastatic cells, with no effect on the primary tumor. Importantly, we demonstrate that IL-6 education of MDPs alone enhances the metastatic potential of tumor cells. Specifically, IL-6-educated MDPs adoptively transferred into met-low tumor-bearing mice, resulting in increased metastasis, in a similar phenotype found in met-high tumors. These results may hold in humans, as we revealed a moderate correlation between IL-6 and gene signature of M2 but not M1 macrophages in human breast cancer samples from the METABRIC data set. Thus, IL-6 should be further tested as a prognostic biomarker for metastasis in the context of immune cell composition in tumors and metastatic sites.

In this study, anti-IL-6 treatment resulted in a dramatic drop in lung metastasis in met-high tumor-bearing mice, whereas no effect was observed in the met-low group. We showed that anti-IL-6 treatment reduced the levels of MDPs in the BM along with M2 macrophages in the lungs of met-high tumor-bearing mice, suggesting that MDP-derived macrophages serve as key promoters of metastasis formation. Anti-IL-6 monoclonal therapy was recently approved by the Food and Drug Administration for patients with Castleman disease.^41^ In the context of solid tumors, despite success in preclinical studies,^41^ the efficacy of such treatment was limited in Phase I–II clinical trials among patients with prostate, ovarian, and lung cancers.^46 47^ Our results further support these clinical studies, because we demonstrated that anti-IL-6 treatment does not inhibit primary tumor growth, whereas it dramatically reduces metastatic burden. In the clinic, anti-IL-6 studies were performed in patients with already advanced metastatic disease. Our study therefore suggests that evaluating plasma levels of IL-6 at early stages, before metastases appear, may provide better insights into the therapeutic potential of IL-6 inhibition. In this regard, we have previously reported that anticancer treatments in addition to their beneficial therapeutic effect may generate protumorigenic and prometastatic biological processes which can increase cancer cell aggressiveness.^48^ This general phenomenon has also been reported clinically. Specifically, neoadjuvant therapy in patients with breast cancer resulted in increased risk for metastasis.^49^ Thus, it is worth studying whether the addition of anti-IL-6 to the neoadjuvant treatment protocol can potentially reduce the risk for metastasis.

In summary, our study demonstrates a novel mechanism underlying direct tumor-mediated dictation of HSPC differentiation and programming of its progeny. This, in turn, becomes an essential process for tumor progression and metastasis. We demonstrate that the cross talk between the tumor and distant BM niche is mediated by IL-6/IL-6Ra signaling, paving the way toward new therapeutic approaches to target metastasis.

## Data Availability

All data relevant to the study are included in the article or uploaded as supplementary information. Single-cell RNA sequencing of hematopoietic stem and progenitor cells data set is included in the manuscript under online supplemental table S1.
