# Supplementary material for: IL-6 contributes to metastatic switch via the differentiation of monocytic-dendritic progenitors into prometastatic immune cells
Source: J Immunother Cancer. 2021 Jun 17;9(6):e002856. doi: 10.1136/jitc-2021-002856 (PMC8212411; doi:10.1136/jitc-2021-002856)
Supplement: Supplementary data [file jitc-2021-002856supp002.pdf]

Supplemental Materials Online

Magidey-Klein et. al.,

Supplemental Table S2: Breast and lung cancer patient characteristics

Sample ID	Age	Gender	Tumor type	Grade	Stage	Early/ Advanced
**Breast cancer**						
308_A_1	62	F	Invasive lobular carcinoma	n/a	II	A
308_A_2	50	F	Invasive ductal carcinoma	G2	II	A
308_E_1	49	F	Invasive ductal carcinoma	G1	IA	E
308_E_2	66	F	Invasive ductal carcinoma	G2	IA	E
308_A_3	44	F	Invasive papillary carcinoma	G2-3	III	A
308_E_3	55	F	Ductal carcinoma in situ	G2	0	E
308_E_4	69	F	Ductal carcinoma in situ	G2-3	0	E
308_E_6	68	F	Ductal carcinoma in situ	G3	0	E
308_A_4	81	F	Invasive lobular carcinoma	G1-2	IIIA	A
308_E_9	65	F	Invasive ductal carcinoma	G2	IA	E
308_A_5	56	F	Invasive lobular carcinoma	G3	III	A
308_E_11	60	F	Invasive ductal carcinoma	G2	IA	E
308_E_12	47	F	Ductal carcinoma in situ	G2	0	E
308_A_6	48	F	Invasive ductal carcinoma	G3	IIA	A
308_E_13	48	F	Invasive ductal carcinoma	G1	IA	E
308_A_8	78	F	Infiltrating ductal carcinoma	G2	IIB	A
308_A_9	73	F	Invasive ductal carcinoma	n/a	IV	A
308_A_10	38	F	Invasive ductal carcinoma	G3	IIB	A
**Lung cancer**						
297A_A_10	73	F	Papillary adenocarcinoma	G3	IV	A
297A_E_6	59	F	Mucinous adenocarcinoma	G1	IA	E
297A_E_3	67	M	Papillary adenocarcinoma	n/a	IIA	E
297A_A_3	48	M	Papillary adenocarcinoma	n/a	IIIA	A
297A_A_3.1	48	M	Papillary adenocarcinoma	n/a	IIIA	A
297A_A_4	53	M	Clear cell carcinoma	n/a	IV	A
297A_E_5	69	F	Adenocarcinoma in situ	n/a	0	E
297A_A_9	80	M	Adenocarcinoma (acinar and solid)	n/a	IIIA-IV	A
297A_A_5	85	F	Squamous cell carcinoma	G3	IIIB	A
297A_A_6	71	M	Squamous cell carcinoma	G3	IIIB	A
297A_A_7	74	M	Adenocarcinoma	G3	IIIA	A
297A_E_8	61	M	Adenocarcinoma in situ	n/a	0	E
297A_E_9	63	F	Adenocarcinoma in situ	n/a	0	E
297A_E_10	77	F	Adenocarcinoma	G1	IA	E
297A_A_8	60	F	Adenocarcinoma (acinar and solid)	n/a	IIIA	A
297_E_1	68	F	Adenocarcinoma	G1	IA	E
297_E_2	70	F	Adenocarcinoma (acinar)	G1	IA	E

Magidey-Klein K, *et al. J Immunother Cancer* 2021; 9:e002856. doi: 10.1136/jitc-2021-002856

Supplemental Table S3: Phenotype characteristics of immune cells in murine and human.

Population	Markers
**In murine**	
LSK	Lineage-Sca-1+CD117+
GMP	Lineage-Sca-1-CD117+CD34-FcγR+
CMP	Lineage-Sca-1-CD117+CD34+FcγR-
MEP	Lineage-Sca-1-CD117+CD34- FcγR-
MDP	Lineage-Sca-1-CD117+CD34+FcγR-CD115+FLT3+
Granulocytes	CD11b+Ly6G+/Gr-1+
Macrophages	Cd11b+F4/80+Ly6G-
M1 macrophages	Cd11b+F4/80+CD11c+Ly6G-
M2 macrophages	CD11b+ F4/80+CD206+Ly6G-
Inflammatory monocytes	CD11b+Ly6C^{high}Ly6G^{low} CD115+
Anti-inflammatory monocytes	CD11b+Ly6C^{low}Ly6G-
Dendritic cells	Cd11b+F4/80+Ly6G-MHCII+
B cells	CD45+B220+
T cells	CD45+CD3ε+
**In human**	
MDP	Lineage-CD34+CD38^{high}CD123^{Int}CD45RA+CD115+
GMP	Lineage-CD34+CD38+CD123+CD34RA+

Magidey-Klein K, *et al. J Immunother Cancer* 2021; 9:e002856. doi: 10.1136/jitc-2021-002856

Supplemental Table S4: Antibodies against surface markers conjugated with metal ions for CyTOF dataset.

Metal	Ab
141Pr	CD80
142Nd	GR1
143Nd	CD86
144Nd	F4/80
145Nd	CD4
146Nd	CD45R
147Sm	Ly6c
148Nd	CD138
149Sm	CD8
150Nd	Ly6g
151Eu	CD48
152Sm	CD90
153Eu	CD14
154Sm	CD11c
155Gd	CD45
156Gd	CD49b
157Gd	CD19
158Gd	CD34
159Tb	CD27
160Gd	CD69
161Dy	CD150
162Dy	TCRb
163Dy	CD127
164Dy	CD28
165Ho	CD115
166Er	CD133
167Er	CD93
168Er	CD117
169Tm	CD79b
170Er	CD62L
171Yb	CD44
172Yb	CD43
173Yb	Sca-1
174Yb	Vegfr2
175Lu	CD5
176Yb	Cd11b

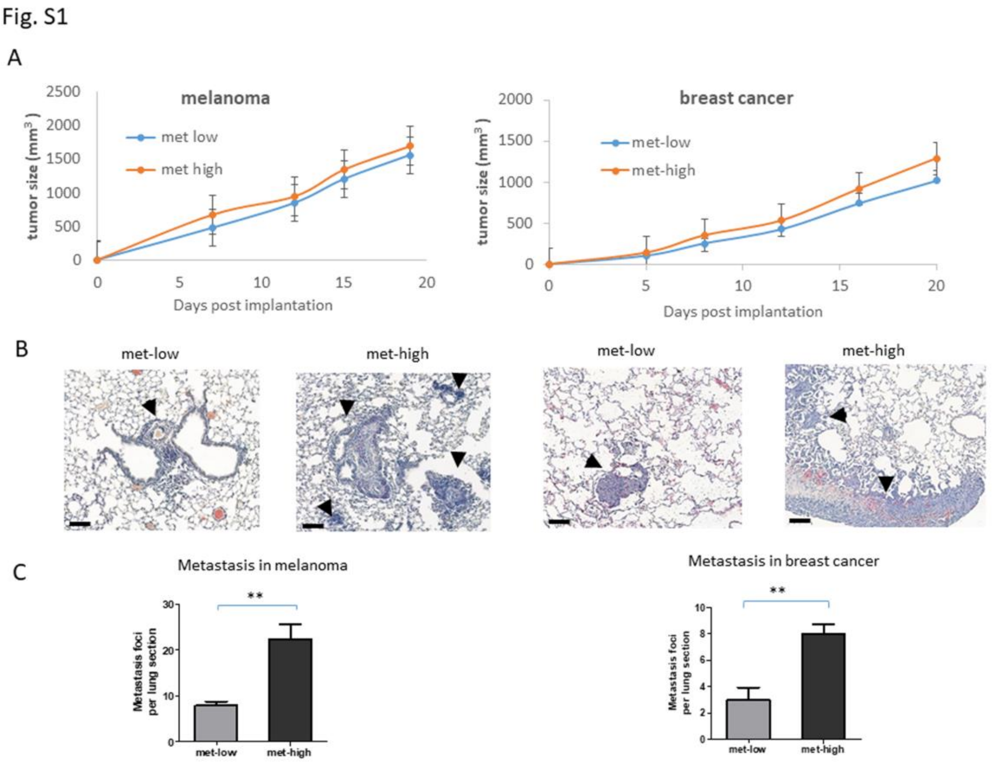

Figure S1. Rates of tumor growth and metastasis in met-high and met-low melanoma and breast tumor models. C57BL/6 mice were sub-dermally implanted in the flank with met-low B16-F1 or met-high B16-F10 melanoma cells. BALB/c mice were implanted in the mammary fat pad with met-low 67NR or met-high 4T1 breast cancer cells (n=5 mice/group). Tumor growth was monitored regularly. At endpoint, lungs were removed and assessed for metastasis. (A) Tumor growth curves are presented. (B) Representative lung sections are shown, bar=100µm. Arrows indicate metastatic foci. (C) Metastatic foci per lung section were quantified (n=5 sections/mouse). Significant p values are shown as ** p<0.01, as assessed by unpaired two-tailed t-test.

Magidey-Klein K, *et al. J Immunother Cancer* 2021; 9:e002856. doi: 10.1136/jitc-2021-002856

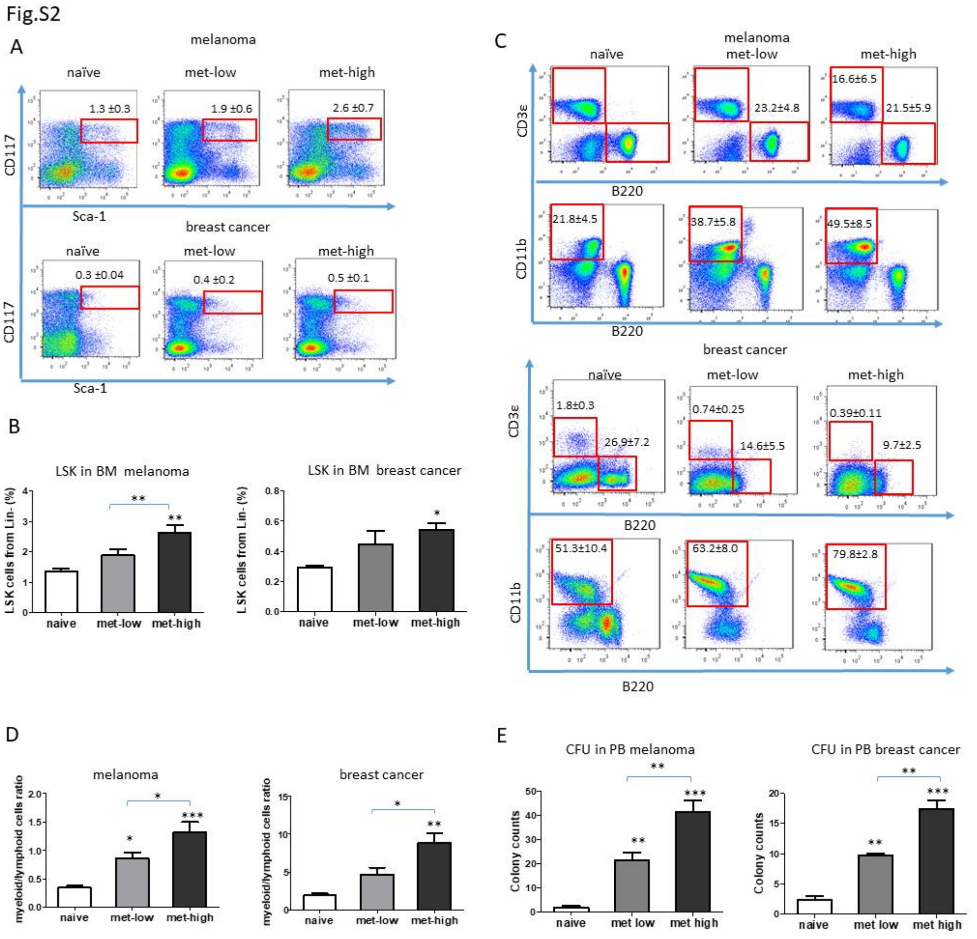

Figure S2. Metastatic tumors induce myelopoiesis. Mice were either sub-dermally implanted in the flank with met-low B16-F1 or met-high B16-F10 melanoma cells (5x10^5 cell/mouse), or implanted in the mammary fat pad with met-low 67NR or met-high 4T1 breast cancer cells (5x10^5 cell/mouse). Control mice (naïve) were not implanted with tumor cells. At endpoint (day 18 and day 21, respectively), mice were sacrificed, and bone marrow and blood were harvested. (A) LSK cells in the BM were analyzed by flow cytometry. The cells were previously gated to exclude doublets and lineage positive cells. Representative flow cytometry plots are shown. (B) Quantification of LSK cells from the lineage negative cells (n=5-8 mice/group). (C) Lymphoid T and B cells and myeloid CD11b+ cells were quantified in the BM by flow cytometry. Representative dot plots are shown (n=5-8 mice/group). (D) Myeloid to lymphoid cell ratios in the bone marrow

Magidey-Klein K, *et al. J Immunother Cancer* 2021; 9:e002856. doi: 10.1136/jitc-2021-002856

compartment are shown (n=5-8 mice/group). (E) Circulating hematopoietic stem and progenitor cells in peripheral blood (PB) were evaluated by a colony forming assay. CFU colony counts are shown (n=3-4 mice/group). Statistical significance was assessed by one-way ANOVA, followed by Tukey post-hoc test when comparing more than two groups or unpaired two-tailed t-test when comparing two groups. Asterisks represent significance from control, unless indicated otherwise in the figure. Significant p values are shown as *, $p<0.05$, ** $p<0.01$, ***$p<0.001$.

Fig. S3

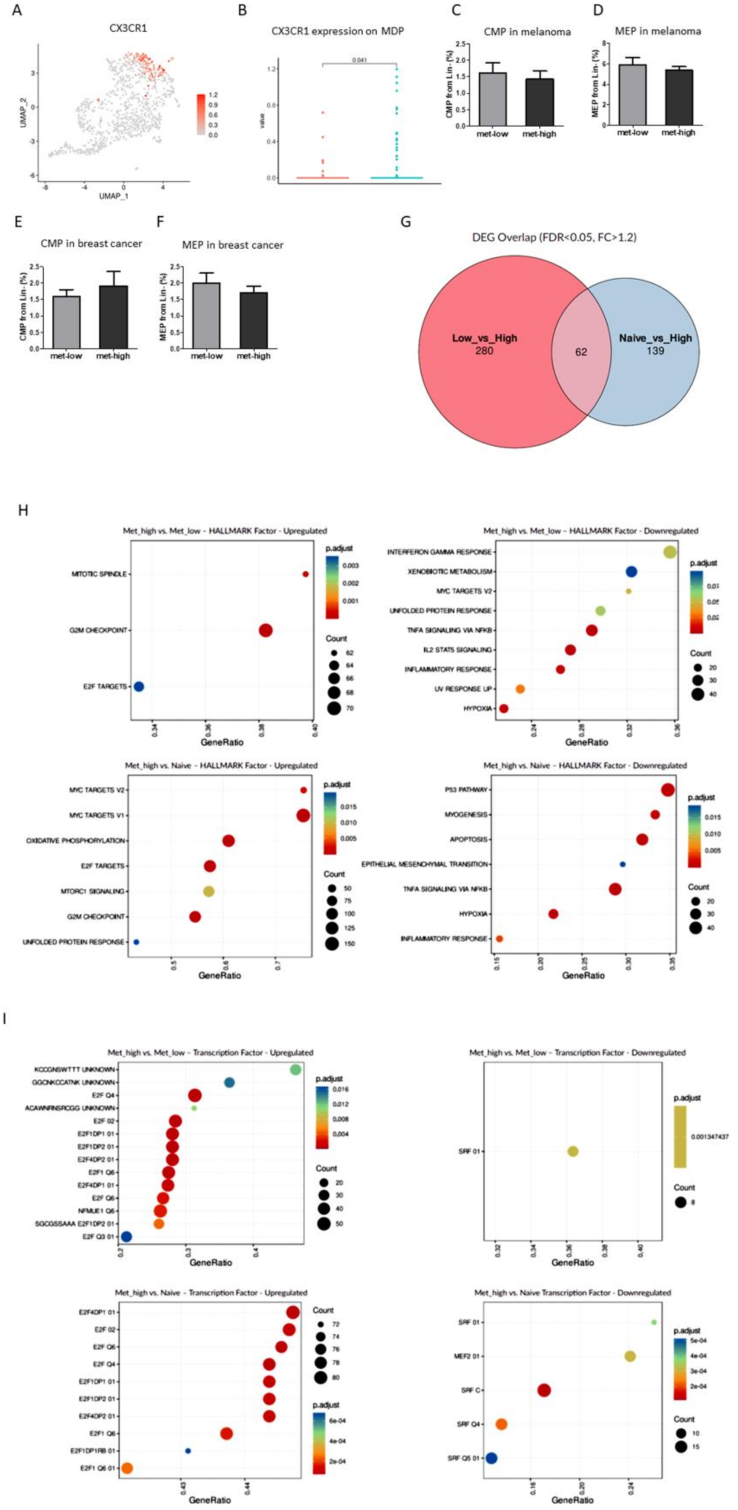

A
CX3CR1
B
CX3CR1 expression on MDP
C
CMP in melanoma
D
MEP in melanoma
E
CMP in breast cancer
F
MEP in breast cancer
G
DEG Overlap (FDR<0.05, FC>1.2)
Low_vs_High
280
62
Naive_vs_High
139
H
Met_high vs. Met_low - HALLMARK Factor - Upregulated
Met_high vs. Met_low - HALLMARK Factor - Downregulated
Met_high vs. Naive - HALLMARK Factor - Upregulated
Met_high vs. Naive - HALLMARK Factor - Downregulated
I
Met_high vs. Met_low - Transcription Factor - Upregulated
Met_high vs. Met_low - Transcription Factor - Downregulated
Met_high vs. Naive - Transcription Factor - Upregulated
Met_high vs. Naive Transcription Factor - Downregulated

Figure S3. LSK clustering in met-high and met-low tumors. Mice were implanted with met-high or met-low melanoma tumor cells. At endpoint, LSK cells were obtained from bone marrow and analyzed by single cell RNA sequencing**.** (A-B) Single cell CX3CR1 mRNA expression labeling on the UMAP plot (A) and its expression specifically in MDP populations of met-low and met-high tumors (B). Statistical significance was assessed by Wilcoxon rank-sum test. (C-F) Flow cytometry validation of CMP (C,E) and MEP (D, F) percentages from Lin- cells of melanoma (C, E) or breast cancer model (D, F) met-low and met-high tumor bearing mice (n=5 mice/group in melanoma model, n=5-10 mice/group in breast cancer model). Results are not statistically significant. (G) Venn diagram of differentially expressed genes (DEGs) in naïve and met-low MDPs vs met-high MDPs. (H) GSEA analysis of upregulated and downregulated biological processes comparing met-low MDPs or naïve MDPs vs met-high MDPs. (I) Transcription factors enrichment in met-low/naïve MDPs comparing with met-low MDPs.

Fig. S4

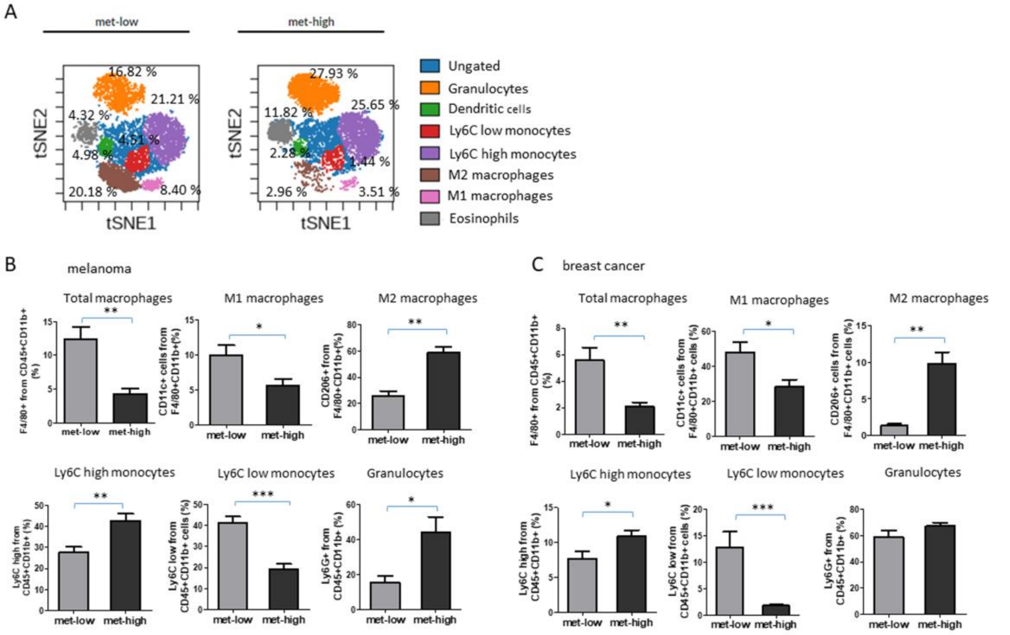

Figure S4. Immune cell composition in met-high and met-low tumors. (A-C) Mice were implanted with met-high or met-low tumor cells of melanoma (A-B) or breast cancer (C). At endpoint, tumors were removed and prepared as single cell suspensions. (A) viSNE plot obtained by immune cell CyTOF analysis is based on CD11b+ myeloid cells of met-low and met-high melanomas (3 samples per group were pooled). (B-C) The percentages of macrophages and their M1 and M2 subsets, Ly6C^{high}, Ly6C^{low} monocytes and granulocytes were analyzed by flow cytometry on melanoma (B) and breast cancer (C) models (n=5-8 mice for each group). Statistical significance was assessed by unpaired two-tailed t-test. Significant p values are shown as * p<0.05; ** p<0.01; *** p<0.001.

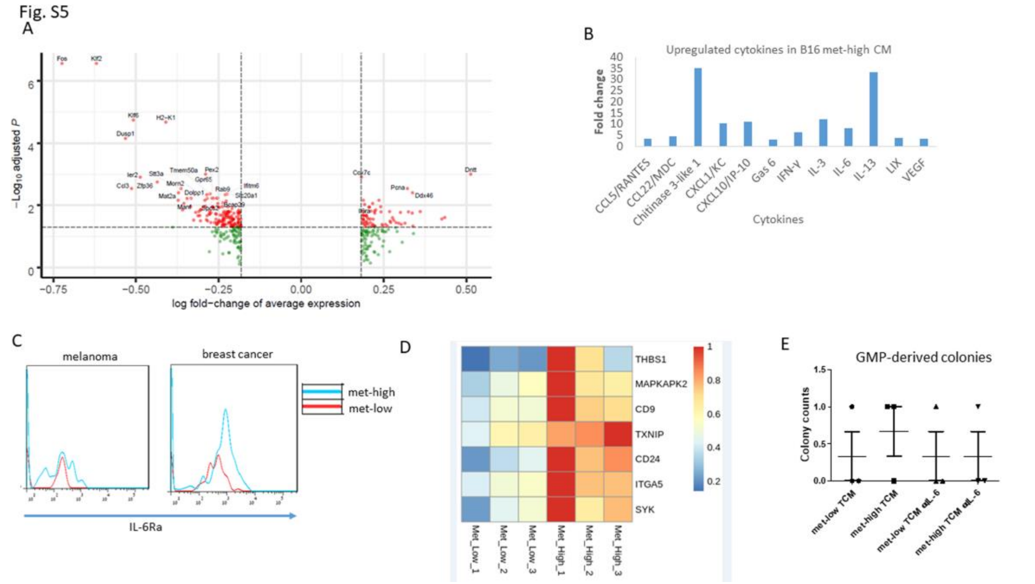

Figure S5. The secretion profile of met-high tumors is associated with IL-6. (A) Volcano plot of significantly decreased (left side) and increased (right side) genes in MDP cells (met-high vs. met-low groups; melanoma model; Wilcoxon rank-sum test followed by Benjamini–Hochberg correction). (B) A cytokine array was performed to determine the levels of a range of factors in tumor conditioned medium (TCM) of met-high and met-low melanomas. Fold change is shown (met-high vs. met-low). (C) Representative histogram plots of IL-6Ra expression on MDPs, obtained from bone marrow of met-low and met-high melanoma or breast cancer bearing mice, was assessed by flow cytometry. (D) Heat map of differentially expressed genes of CD45+ live cells, obtained from met-low and met-high melanoma tumors. (E) GMPs from naïve mice were grown in Methocult medium supplemented with met-low or met-high melanoma tumor conditioned medium (TCM) in the presence or absence of anti-IL-6 neutralizing antibodies. GMP-derived colonies were counted (n=3 biological repeats/group).

Magidey-Klein K, *et al. J Immunother Cancer* 2021; 9:e002856. doi: 10.1136/jitc-2021-002856

Fig. S6

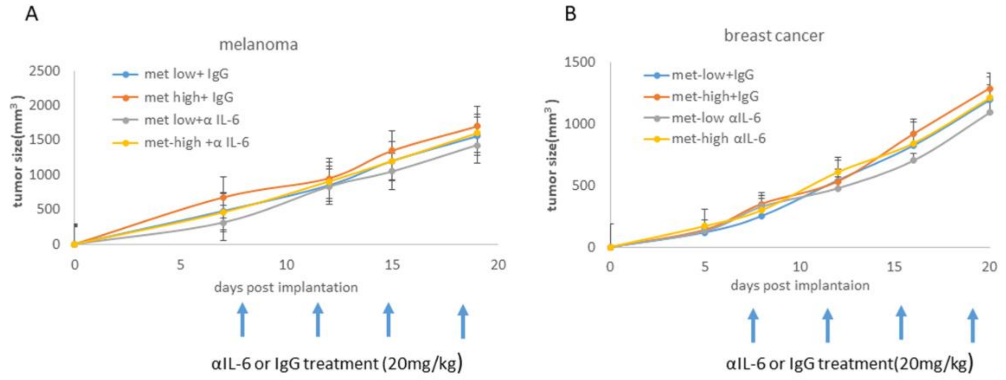

Figure S6. Blocking IL-6 does not affect primary tumor growth. Mice were implanted with met-low or met-high (A) melanoma or (B) breast cancer cells. One week later, mice were treated with IgG (control) or anti-IL-6 antibodies twice weekly (indicated by arrows). Tumor growth curves are shown (n=5-8 mice/group).

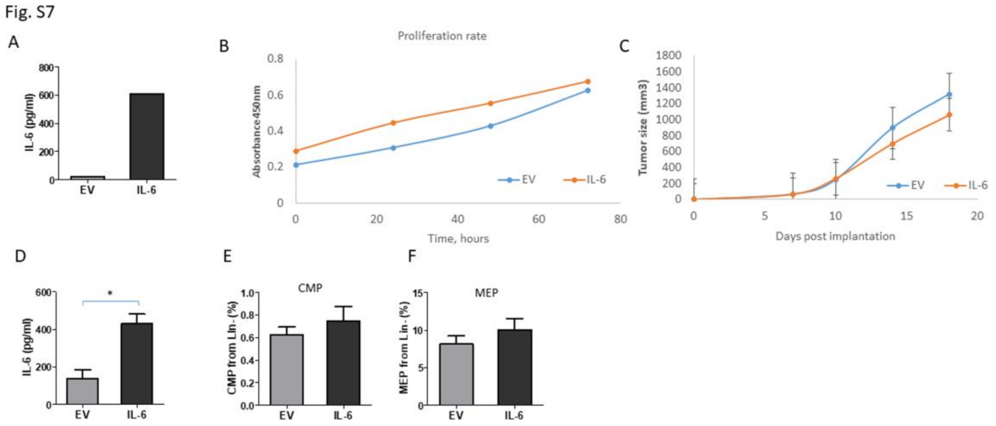

Figure S7. IL-6 overexpression in met-low tumors and its effect on HSPC lineage. (A) Conditioned medium collected from IL-6 overexpressing and EV control B16-F1 cells was tested for IL-6 levels by ELISA. (B) Proliferation rate of IL-6 overexpressing cells and EV control cells was tested by XTT assay. (C-G) Eight-to-ten week old C57Bl/6 mice (n=4-6 mice/group) were implanted with B16-F1-IL-6 overexpressing cells or with corresponding control EV cells. Tumor growth was assessed (C). At endpoint (day 18), mice were sacrificed, tumors were removed and the bone marrow was harvested IL-6 levels were quantified in B16-F1 overexpressing IL-6 and B16-F1 EV control TCM, using ELISA (D). The levels of CMP (E), and MEP (F) from Lin- cells were assessed in the bone marrow of mice from both groups by flow cytometry. Statistical significance was assessed by unpaired two-tailed t-test. Significant p values are shown as * $p<0.05$.

Magidey-Klein K, *et al. J Immunother Cancer* 2021; 9:e002856. doi: 10.1136/jitc-2021-002856
